# DYRK2 downregulation in colorectal cancer leads to epithelial–mesenchymal transition induction and chemoresistance

**DOI:** 10.1038/s41598-022-25053-0

**Published:** 2022-12-28

**Authors:** Chunrong Wu, Guiyin Sun, Fan Wang, Jiangyan Chen, Fangbiao Zhan, Xiaojuan Lian, Jie Wang, Fanbin Weng, Bo Li, Weijun Tang, Jin Quan, Debing Xiang

**Affiliations:** 1 Department of Oncology, Chongqing University Jiangjin Hospital, School of Medicine, Chongqing University, Chongqing, 402260 China; 2 Department of Oncology, Jiangjin Central Hospital of Chongqing, Chongqing, 402260 China; 3 Department of Orthopedics, Chongqing University, Three Gorges Hospital, Wanzhou, Chongqing, 404000 China; 4 Department of Cardiology, Chongqing University Jiangjin Hospital, School of Medicine, Chongqing University, Chongqing, 402260 China

**Keywords:** Cancer, Cell biology, Molecular biology, Diseases, Oncology

## Abstract

Colorectal cancer (CRC) is among the most prominent causes of cancer-associated mortality in the world, with chemoresistance representing one of the leading causes of treatment failure. However, the mechanisms governing such chemoresistance remain incompletely understood. In this study, the role of DYRK2 as a mediator of CRC cell drug resistance and the associated molecular mechanisms were assessed by evaluating human tumor tissue samples, CRC cell lines, and animal model systems. Initial analyses of The Cancer Genome Atlas database and clinical tissue microarrays revealed significant DYRK2 downregulation in CRC in a manner correlated with poor prognosis. We further generated LoVo CRC cells that were resistant to the chemotherapeutic drug 5-FU, and found that such chemoresistance was associated with the downregulation of DYRK2 and a more aggressive mesenchymal phenotype. When DYRK2 was overexpressed in these cells, their proliferative, migratory, and invasive activities were reduced and they were more prone to apoptotic death. DYRK2 overexpression was also associated with enhanced chemosensitivity and the inhibition of epithelial–mesenchymal transition (EMT) induction in these LoVo 5-FUR cells. Co-immunoprecipitation assays revealed that DYRK2 bound to Twist and promoted its proteasomal degradation. In vivo studies further confirmed that the overexpression of DYRK2 inhibited human CRC xenograft tumor growth with concomitant Twist downregulation. Overall, these results thus highlight DYRK2 as a promising therapeutic target in CRC worthy of further investigation.

## Introduction

Colorectal cancer (CRC) is among the most common and deadliest forms of cancer, with individuals suffering from advanced and metastatic disease exhibiting particularly high mortality rates^1^. Treatment approaches for CRC include combinations of surgical, chemotherapeutic, radiological, and targeted interventions, with chemotherapy being a cornerstone treatment strategy that is universally employed. At present, 5-fluorouracil (5-FU) remains one of the most commonly used and important chemotherapeutic drugs used to treat CRC^2,3^, functioning by irreversibly inhibiting thymidylate synthase, resulting in the impairment of DNA synthesis^4^. Therapeutic resistance to 5-FU thus represents a major barrier to the effective systemic chemotherapeutic treatment of CRC patients.

The epithelial–mesenchymal transition (EMT) is a process whereby cells shed their epithelial characteristics and acquire mesenchymal characteristics through a series of conserved mechanisms^5^. Epithelial cells lose their polarization and detach from the basement membrane upon EMT induction, instead acquiring mesenchymal-like properties including enhanced migratory and invasive activity^6^, cancer stem cell characteristics, and apoptotic resistance^7^. Several studies have shown that EMT is associated with the emergence of chemotherapeutic resistance^8,9^. A range of important signaling pathways govern EMT induction and progression, including the integrin/EGFR-ERK/MAPK signaling pathway^10^, the Wnt and Notch signaling pathways^11^, transforming growth factor β (TGF-β)^12^, endothelin A receptor (ETAR), matrix metalloproteinases (MMPs), and hypoxia^13^.

Dual-specificity tyrosine phosphorylation-regulated kinase 2 (DYRK2) is an important regulator of DNA damage repair, cell cycle progression, and apoptotic cell death^14,15^. Multiple studies show that DYRK2 has been shown to control EMT induction in a range of tumor types^16,17^. Recent bioinformatics analysis suggest that DYRK2 is the only kinase found to be significantly downregulated in CRC^18^. Moreover, recent evidence suggests that DYRK2 downregulation was closely associated with liver metastasis and patient prognosis^19,20^. However, it remains unclear whether DYRK2 is involved in the regulation of chemotherapy resistance in CRC and whether it can thereby affect organ metastasis and the prognosis of CRC patients.

As such, in the present study we sought to conduct an in-depth analysis of the role of DYRK2 as a mediator of CRC cell chemoresistance, with a specific focus on its ability to regulate EMT induction through analyses of human tumor tissue samples, CRC cells, and animal model systems. Together, these results will provide a novel foundation for future efforts to treat CRC.

## Results

### DYRK2 is downregulated in CRC and associated with patient prognosis and clinicopathological characteristics

**Figure 1:**
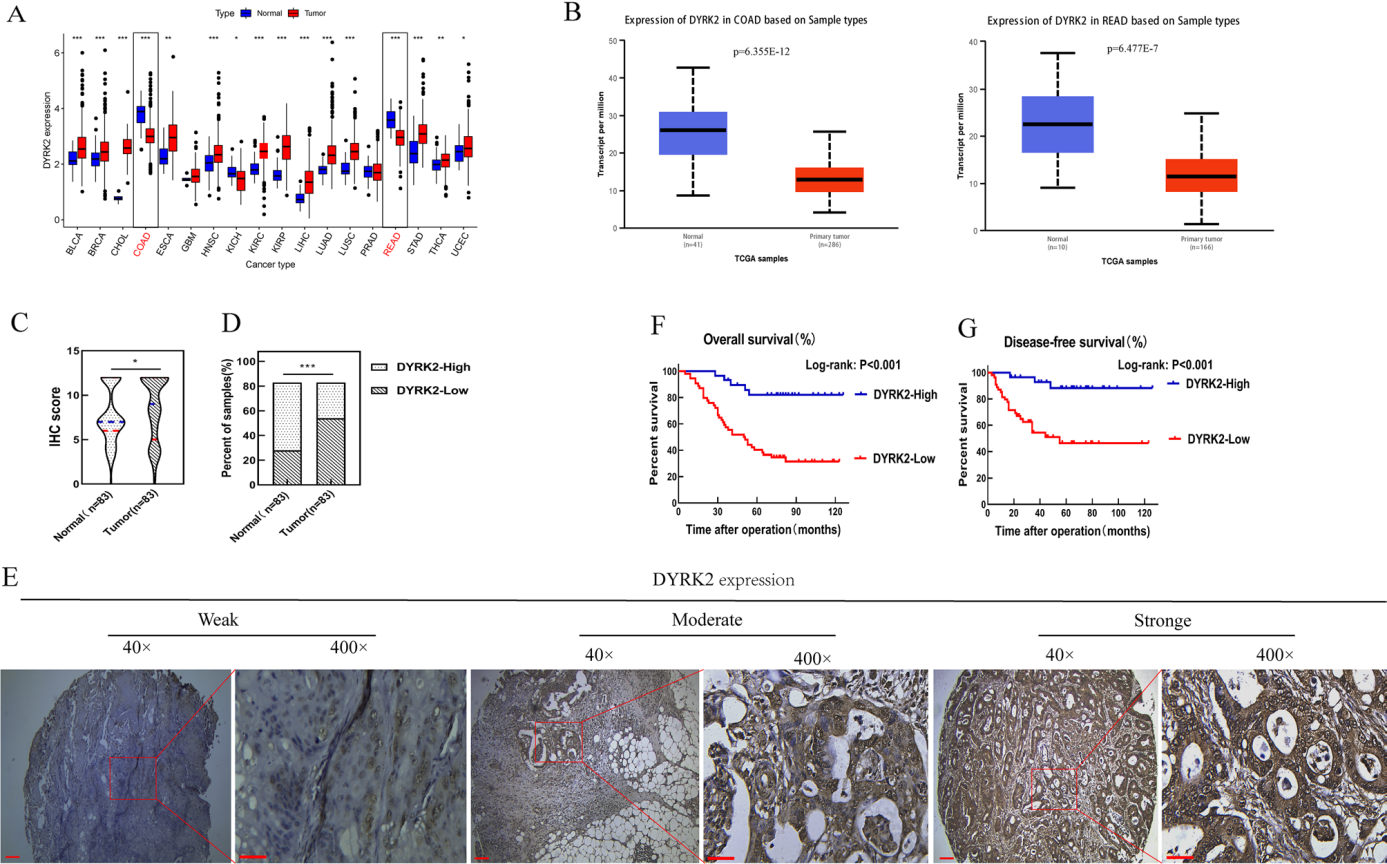
Analysis of the clinicopathological correlates of DYRK2 expression in CRC. (**A**) Pan-cancer DYRK2 mRNA expression profiles in humans were assessed using the TIMER database, revealing DYRK2 upregulation in 13 tumor types (BLCA, BRCA, CHOL, ESCA, HNSC, KIRC, KIRP, LIHC, LUAD, LUSC, STAD, THCA, UCEC), and downregulation in 3 tumor types (COAD, KICH, READ), RNA-Seq was conducted via expectation maximization. *BLCA* bladder urothelial carcinoma, *BRCA* breast invasive carcinoma, *CHOL* cholangio carcinoma, *ESCA* esophageal carcinoma, *HNSC* head and neck squamous cell carcinoma, *KIRC* kidney renal clear cell carcinoma, *KIRP* kidney renal papillary cell carcinoma, *LIHC* liver hepatocellular carcinoma, *LUAD* lung adenocarcinoma, *LUSC* lung squamous cell carcinoma, *STAD* stomach adenocarcinoma, *THCA* thyroid carcinoma, *UCEC* uterine corpus endometrial carcinoma, *COAD *colon adenocarcinoma, *KICH* kidney chromophobe, *READ* rectal adenocarcinoma. (**B**) DYRK2 expression levels were assessed in CRC tumors and paracancerous tissues using TCGA datasets. *COAD* colon adenocarcinoma, *READ* rectal adenocarcinoma. (**C**,**D**) DYRK2 protein levels were analyzed via IHC staining of TMAs containing 83 paired colon carcinoma and healthy paracancerous tissue samples (COC1602). (**E**) Representative images of IHC staining for DYRK2. Note: Scale bar = 100 μm (× 40) or 20 μm (× 400). (**F**,**G**) The relationship between DYRK2 expression and CRC patient survival. The OS and DFS of CRC patients in the TMA-CRC cohort are represented with Kaplan–Meier curves. *P < 0.05, **P < 0.01, ***P < 0.001.

Initial analyses of the Tumor Immune Estimation Resource (TIMER) database analysis were used to explore the expression of DYRK2 at the mRNA level in a range of cancers (Fig. 1A). Further analyses of the TCGA database (http://ualcan.path.uab.edu) revealed pronounced DYRK2 downregulation in CRC tumor tissues relative to the levels in adjacent healthy tissues (Fig. 1B). CRC patient tissue microarrays (TMAs) were further employed to explore the correlative relationships between the expression of DYRK2 and patient clinicopathological characteristics using an immunohistochemical (IHC) staining approach, revealing that 65.06% (54/83) of the samples in this TMA specimens in TMA exhibited low levels of DYRK2 expression, whereas low-level DYRK2 expression was only evident in 34.94% (29/83) of the adjacent paracancerous tissue samples (Fig. 1C,D). More advanced tumor differentiation was additionally associated with DYRK2 downregulation in these CRC tissue samples (Fig. 1E).

To further explore the clinical implications of the observed changes in DYRK2 expression, we next conducted an analysis of the relationship between such expression and CRC patient follow-up outcomes. DYRK2 expression was found to be correlated with CRC patient lymph node metastasis, liver metastasis, and pathological differentiation in these analyses (Table 1). Moreover, among the 54 patients exhibiting low levels of DYRK2 expression, 21 developed liver metastases. There was a significant correlation between DYRK2 levels and both disease-free survival (DFS) and overall survival (OS) in this patient cohort (Fig. 1F,G). These data thus highlight DYRK2 as a prognostic indicator associated with CRC patient clinical outcomes.

**Table 1:** Correlation between DYRK2 expression and clinicopathologic characteristics in patients with CRC.

Characteristic	No. of cases (n = 83)	DYRK2 expression		*P* value^a^
		High (n = 29)	Low (n = 54)	
**Age (y)**				0.653
< 58	40 (48.2)	13 (32.5)	27 (67.5)	
≥ 58	43 (51.8)	16 (37.2)	27 (62.8)	
**Gender**				0.274
Male	44 (53.0)	13 (29.5)	31 (70.5)	
Female	39 (47.0)	16 (41.0)	23 (59.0)	
**Diameter of tumor (cm)**				0.991
< 5	43 (51.8)	15 (34.9)	28 (65.1)	
≥ 5	40 (48.2)	14 (35.0)	26 (65.0)	
**CEA (ng/mL)**				0.091
< 6	41 (49.4)	18 (43.9)	23 (56.1)	
≥ 6	42 (50.6)	11 (26.2)	31 (73.8)	
**CA19-9 (U/mL)**				0.002**
< 18	41 (49.4)	21 (51.2)	20 (48.8)	
≥ 18	42 (50.6)	8 (19.0)	34 (81.0)	
**Pathological grading**				0.025*
pG1	10 (12.0)	7 (70.0)	3 (30.0)	
pG2	53 (63.9)	18 (34.0)	35 (66.0)	
pG3	20 (24.1)	4 (20.0)	16 (80.0)	
**Primary tumor**				0.642
T1, T2	20 (24.1)	7 (35.0)	13 (65.0)	
T3	44 (53.0)	17 (38.6)	27 (61.4)	
T4	19 (22.9)	5 (26.3)	14 (73.7)	
**Lymph node status**				0.009**
N0	50 (60.2)	23 (46.0)	27 (54.0)	
N1–N2	33 (39.8)	6 (18.2)	27 (81.8)	
**TNM stage**				< 0.001***
I	10 (12.0)	7 (70.0)	3 (30.0)	
II	26 (31.3)	16 (61.5)	10 (38.5)	
III	26 (31.3)	6 (23.1)	20 (76.9)	
IV	21 (25.3)	0 (0)	21 (100.0)	
**Liver metastasis**	21 (25.3)	0 (0.0)	21 (100.0)	< 0.001***

^a^χ^2^ test. *P < .05. **P < .01. ***P < .001.

### DYRK2 downregulation is associated with enhanced 5-FUR LoVo cell malignancy

**Figure 2:**
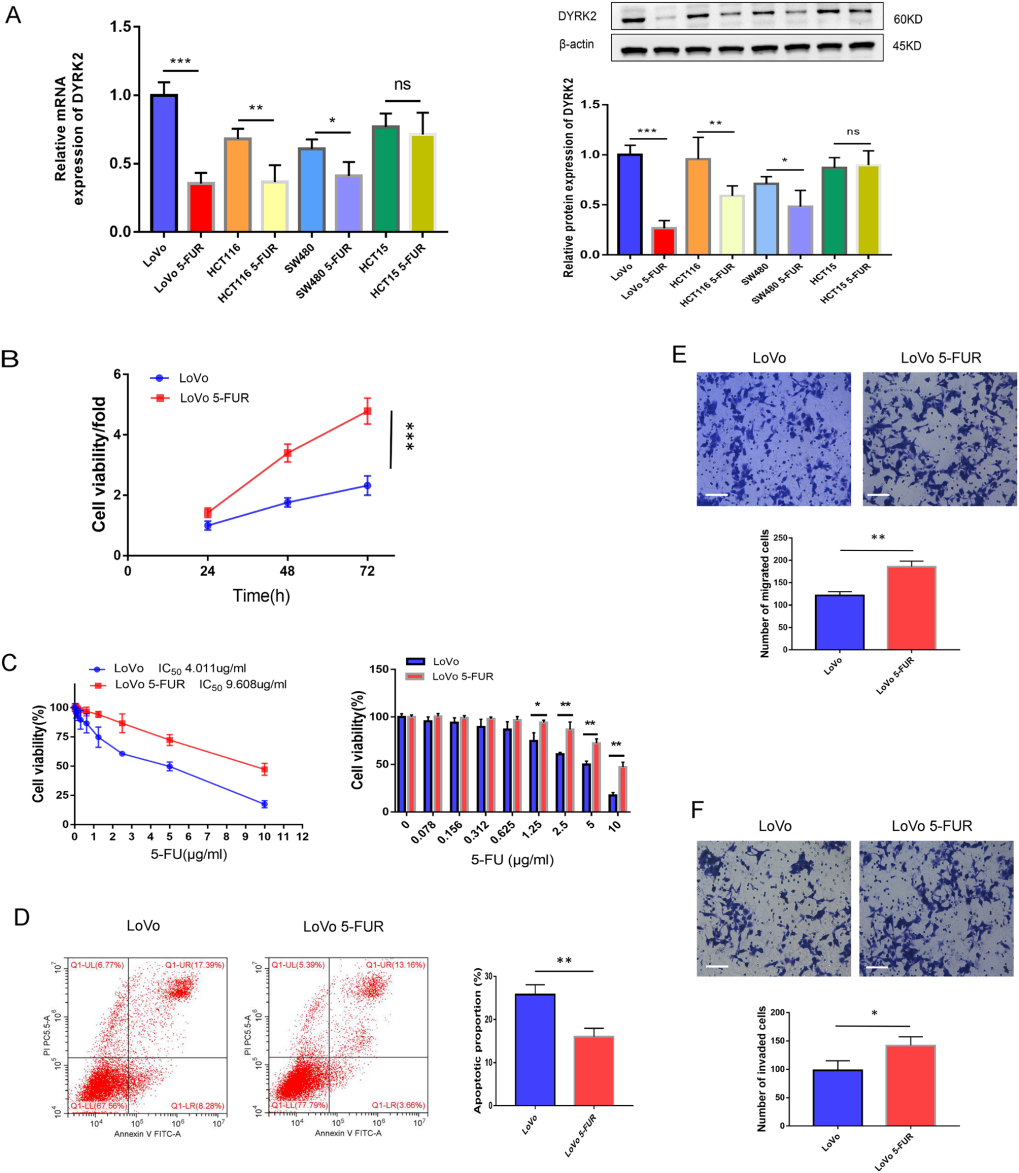
Low levels of DYRK2 expression are associated with enhanced malignancy in 5-FUR LoVo cells. (**A**) Western blotting and qPCR were performed to detect DYRK2 expression in different CRC lines and 5-FU resistant cells. (**B**–**D**) Cellular proliferation, chemosensitivity, and apoptotic death were assessed for both LoVo and 5-FUR LoVo cells via CCK-8 assays and flow cytometry. (**E**,**F**) LoVo and 5-FUR LoVo cell migratory and invasive activity levels were assessed via Transwell assays. Scale bar = 50 μm (× 200). Data are expressed as mean ± SD (n = 3). *P < 0.05, **P < 0.01, ***P < 0.001.

We further explored the expression of DYRK2 in different colorectal cell lines and 5-FU resistant cells (5-FUR). Compared with the parental cells LoVo, HCT116, SW480 and HCT15, we observed that DYRK2 was low expressed in LoVo 5-FUR, HCT116 5-FUR and SW480 5-FUR cell lines both at the mRNA and WB level. However, DYRK2 was not differentially expressed in HCT15 and HCT15 5-FUR cells (Fig. 2A). It was obviously that DYRK2 was significantly decreased in LoVo 5-FUR as compared to parental LoVo cells. Next, we explored the potential role of DYRK2 as a regulator of malignant behaviors in 5-FUR LoVo cells. CCK-8 assays further indicated that 5-FUR LoVo cell viability was enhanced relative to the parental cell line (Fig. 2B). The 5-FU half‐maximal inhibitory concentration (IC50) values for LoVo and 5-FUR LoVo cells were 4.011 μg/mL and 9.608 μg/mL, respectively, consistent with the observed increase in 5-FU resistance in the latter cell line (Fig. 2C). Flow cytometry analyses further revealed decreased rates of apoptosis for 5-FUR LoVo cells (Fig. 2D), while Transwell assays revealed that their migratory and invasive activity was enhanced as compared to parental cells (Fig. 2E,F). Together, these data indicated that DYRK2 downregulation was associated with enhanced malignancy in 5-FUR LoVo cells.

### Low levels of DYRK2 expression are associated with changes in the epithelial–mesenchymal phenotype

**Figure 3:**
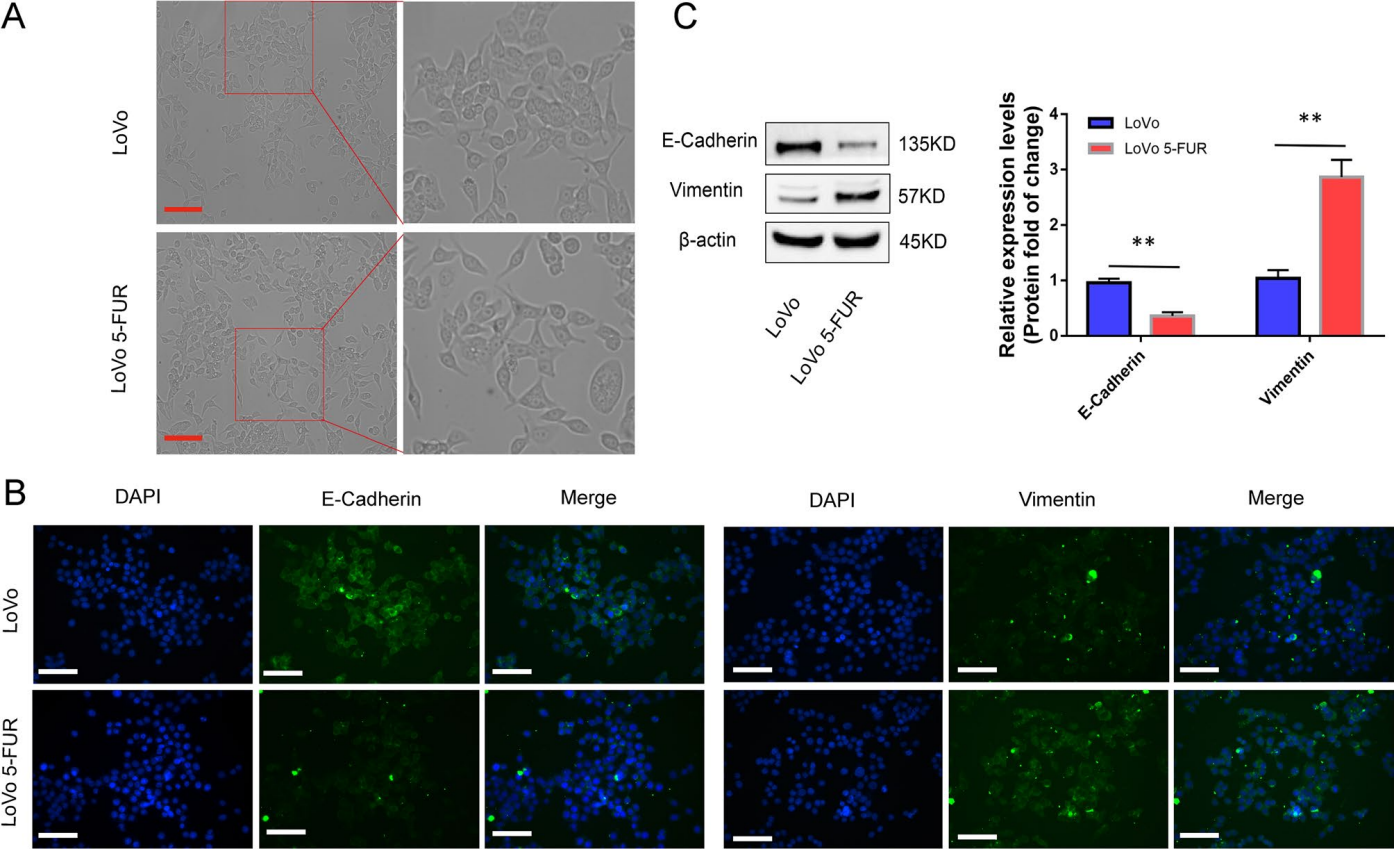
Low levels of DYRK2 expression are associated with changes in CRC cell epithelial–mesenchymal phenotype. (**A**,**B**) Changes in cellular morphology and EMT marker localization. (**A**) Light microscopy was used to assess cellular morphology in a monolayer. Scale bar: 50 μm (× 200). (**B**) E-cadherin and Vimentin localization in LoVo and 5-FUR LoVo cells was assessed via immunofluorescent staining for DAPI (blue) and secondary antibodies (green). Scale bar: 50 μm (× 200). (**C**) EMT marker protein levels were detected via Western blotting and assessed with ImageJ. β-Actin served as a loading control. Data are means ± SD (n = 3). **P < 0.01.

Morphological analyses revealed clear changes in the morphological characteristics of 5-FUR LoVo cells, which exhibits spindle-shaped morphology and irregular, scattered spacing consistent with a mesenchymal phenotype distinct from that observed for parental LoVo cells (Fig. 3A). To confirm these results, we additionally conducted immunofluorescent staining for EMT marker proteins including Vimentin and E-cadherin. Parental cells exhibited the expression of the epithelial marker E-cadherin, and the mesenchymal marker Vimentin was primarily expressed in 5-FUR LoVo cells. E-cadherin expression levels were also lower in these 5-FUR cells as compared to parental LoVo cells, whereas the opposite trend was observed for Vimentin (Fig. 3B). Western blotting further confirmed these results (Fig. 3C). These data thus suggested that EMT induction be associated with the acquisition of chemoresistance following prolonged 5-FU exposure.

### DYRK2 overexpression inhibits 5-FU resistance, proliferation, migration, invasion, and apoptotic resistance in 5-FUR LoVo cells

**Figure 4:**
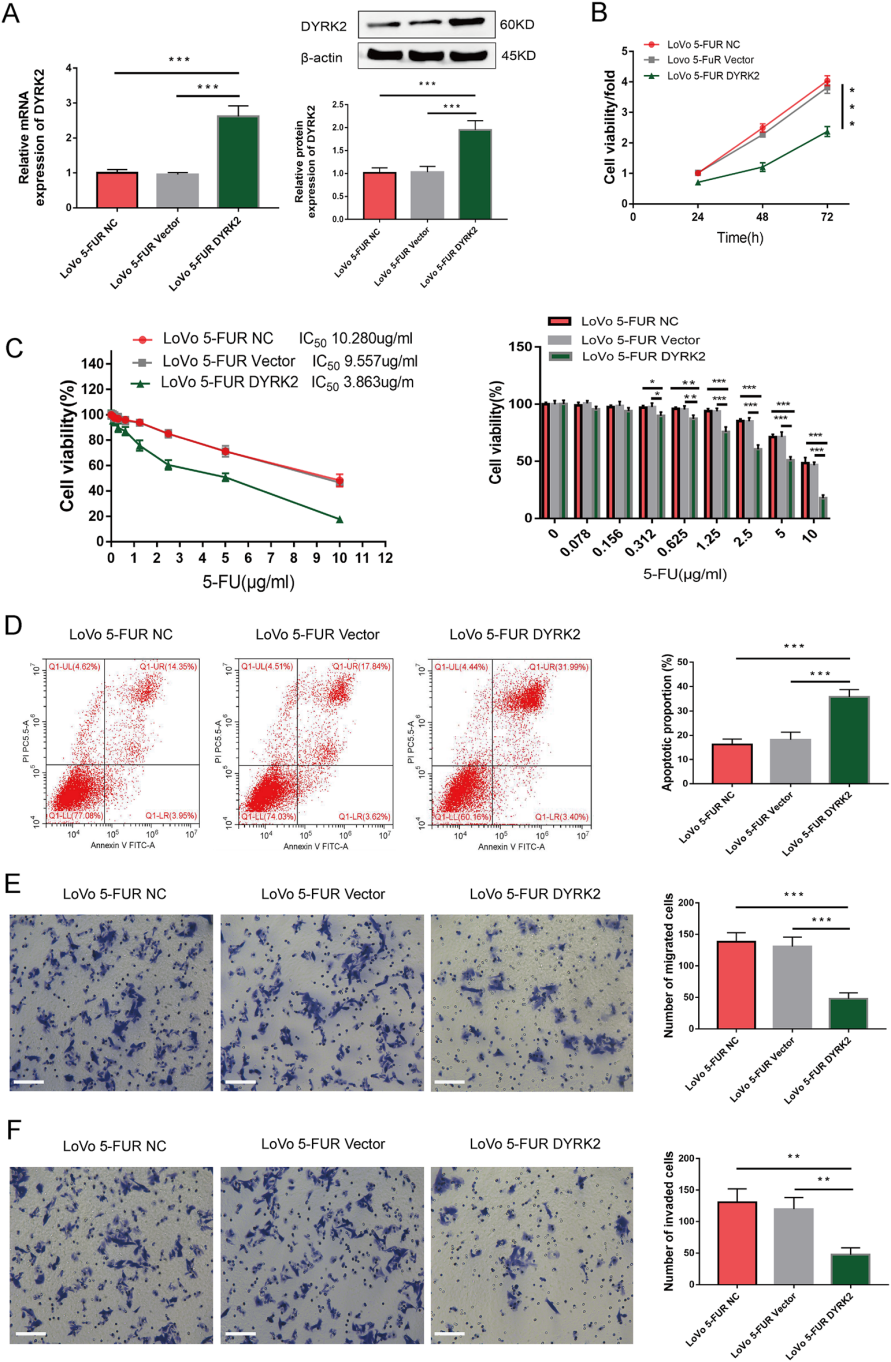
DYRK2 overexpression enhances 5-FUR LoVo cell chemosensitivity and apoptotic death while inhibiting migratory, proliferative, and invasive activity. (**A**) DYRK2 expression in the indicated cells was assessed via qPCR and Western blotting. (**B**,**C**) A CCK-8 assay was used to assess cell viability and chemosensitivity. (**D**) Apoptosis was detected via flow cytometry. Migration (**E**) and invasion assays (**F**) were used to detect the malignant properties of the indicated cells. Scale bar: 50 μm (× 200). Data are means ± SD (n = 3). *P < 0.05, **P < 0.01, ***P < 0.001.

To directly test the ability of DYRK2 to regulate 5-FUR LoVo cell malignancy, these cells were next transduced with DYRK2 overexpression lentiviral vectors or empty control vectors, with qPCR and Western blotting confirming the successful overexpression of DYRK2 in these 5-FUR LoVo cells (Fig. 4A). In subsequent CCK-8 assays, DYRK2 overexpression was associated with impaired viability (Fig. 4B). Moreover, DYRK2 overexpression significantly enhanced the sensitivity of these cells to 5-FU treatment over a 24 h period, with respective IC50 values of 10.280 μg/mL, 9.557 μg/mL, and 3.863 μg/mL for LoVo 5-FUR NC, LoVo 5-FUR Vector, and LoVo 5-FUR DYRK2 (Fig. 4C). The overexpression of this gene additionally induced apoptotic death in these cells while impairing their ability to migrate and engage in invasive activity in Transwell assays (Fig. 4D–F). These results were thus consistent with the function of DYRK2 as a tumor suppressor gene in CRC.

### DYRK2 overexpression restores the epithelial phenotype of 5-FUR LoVo cells

**Figure 5:**
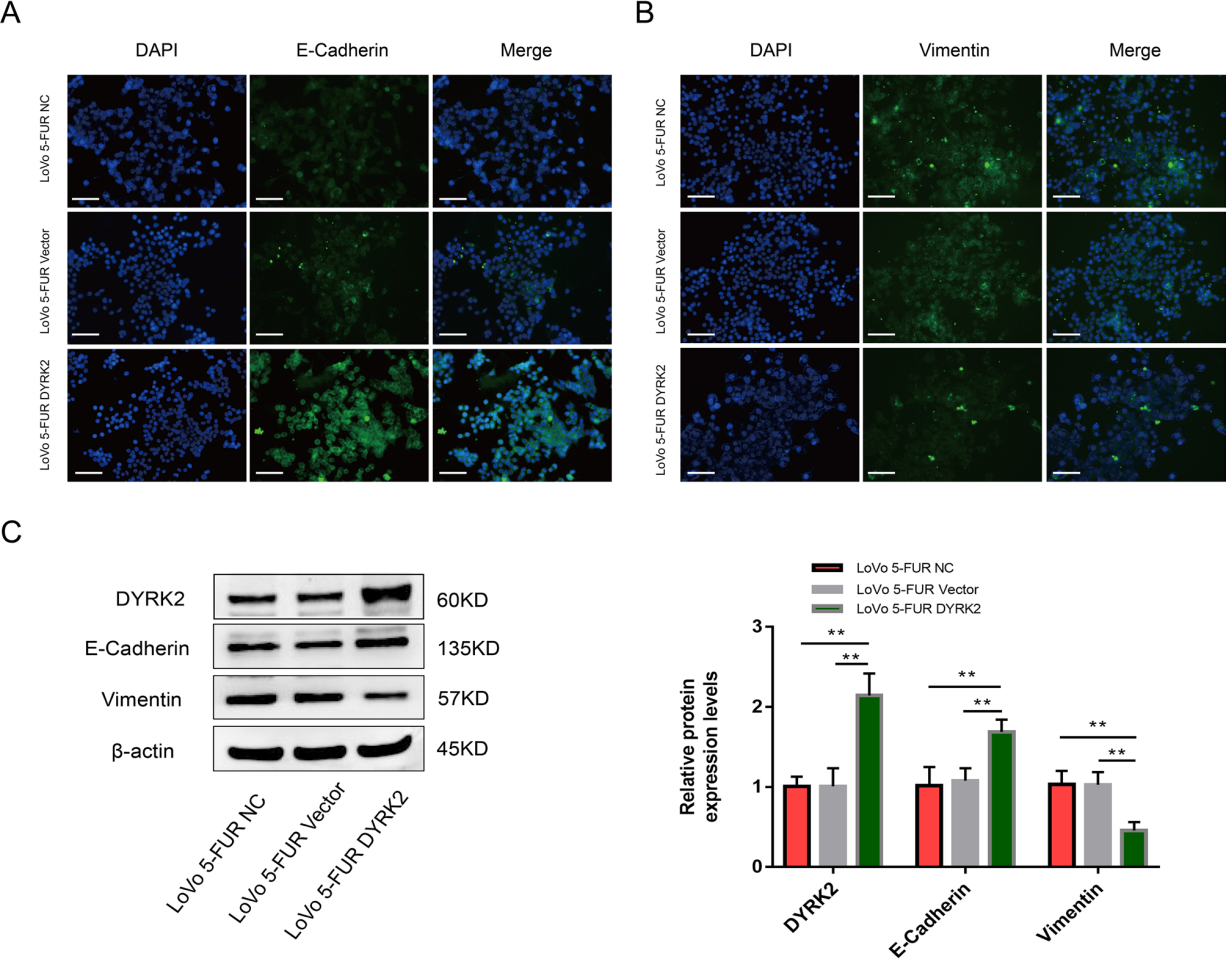
DYRK2 overexpression restores the epithelial phenotype of 5-FUR LoVo cells. (**A**,**B**) DAPI (blue) and secondary antibodies (green) were used for cellular staining. Scale bar: 50 μm (× 200). (**C**) DYRK2 and EMT marker proteins were detected via Western blotting. β-Actin served as a loading control. Data are means ± SD (n = 3). **P < 0.01.

Immunofluorescent staining was next used to assess the expression of E-cadherin and Vimentin in these cells (Fig. 5A,B). Western blotting indicated that the overexpression of DYRK2 resulted in the impairment of EMT induction, as reflected by Vimentin downregulation and E-cadherin upregulation (Fig. 5C).

### Twist is a downstream target gene of DYRK2 in CRC

**Figure 6:**
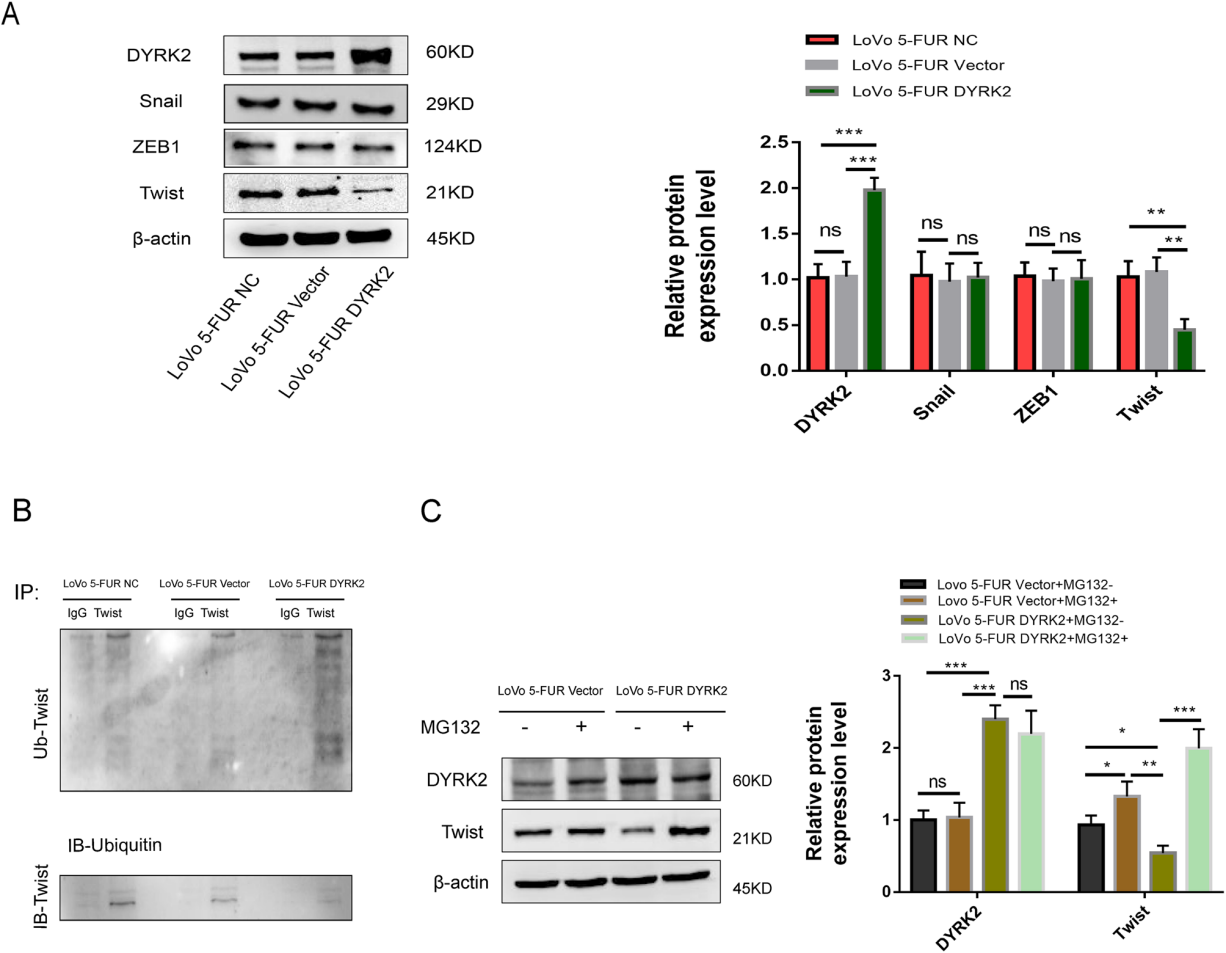
DYRK2 promotes ubiquitin-mediated proteasomal Twist degradation. (**A**) Western blotting was used to assess DYRK2, Snail, ZEB1 and Twist levels in LoVo 5-FUR cells transfected with DYRK2 overexpression or control lentiviruses. (**B**) Anti-Twist and anti-ubiquitin were used for immunoprecipitation. (**C**) LoVo5-FUR cells transfected with DYRK2 overexpression or control vectors were treated for 4 h with MG-132 (10 μM), after which Western blotting was used to assess DYRK2 and Twist protein levels. *P < 0.05, **P < 0.01, ***P < 0.001, *ns* not significant.

In previous reports, common EMT-transcription factors such as Twist, Snail and ZEB have been extensively studied in a range of tumors^21^. To further interrogate the mechanistic link between DYRK2 and EMT induction in CRC cells, we thus analyzed the levels of Twist, ZEB1 and Snail following DYRK2 upregulation. Snail and ZEB1 were not significantly differentially expressed among the three groups, while Twist was highly expressed in the LoVo5-FUR NC and LoVo 5-FUR Vector group, and expressed at relatively low levels in the LoVo 5-FUR DYRK2 group (Fig. 6A).

Therefore, it was potential identity of Twist as a DYRK2 substrate, because reduced Twist protein levels were observed in 5-FUR LoVo cells following the overexpression of DYRK2. To test the ability of DYRK2 to modulate the ubiquitination of Twist, Twist and ubiquitin were co-incubated, revealing Twist ubiquitylation in the context of DYRK2 overexpression consistent with the ability of DYRK2 to promote the ubiquitin modification of Twist (Fig. 6B). The proteasome inhibitor MG132 was then added to cellular lysates to explore the mechanisms governing the degradation of Twist. Twist protein levels decreased rapidly in the presence of DYRK2, whereas these levels were stabilized in the context of MG132 treatment (Fig. 6C). These data thus suggest that DYRK2 can interact with Twist, thereby promoting its proteasomal degradation.

### DYRK2 overexpression inhibits Twist expression and CRC xenograft tumor growth in vivo

**Figure 7:**
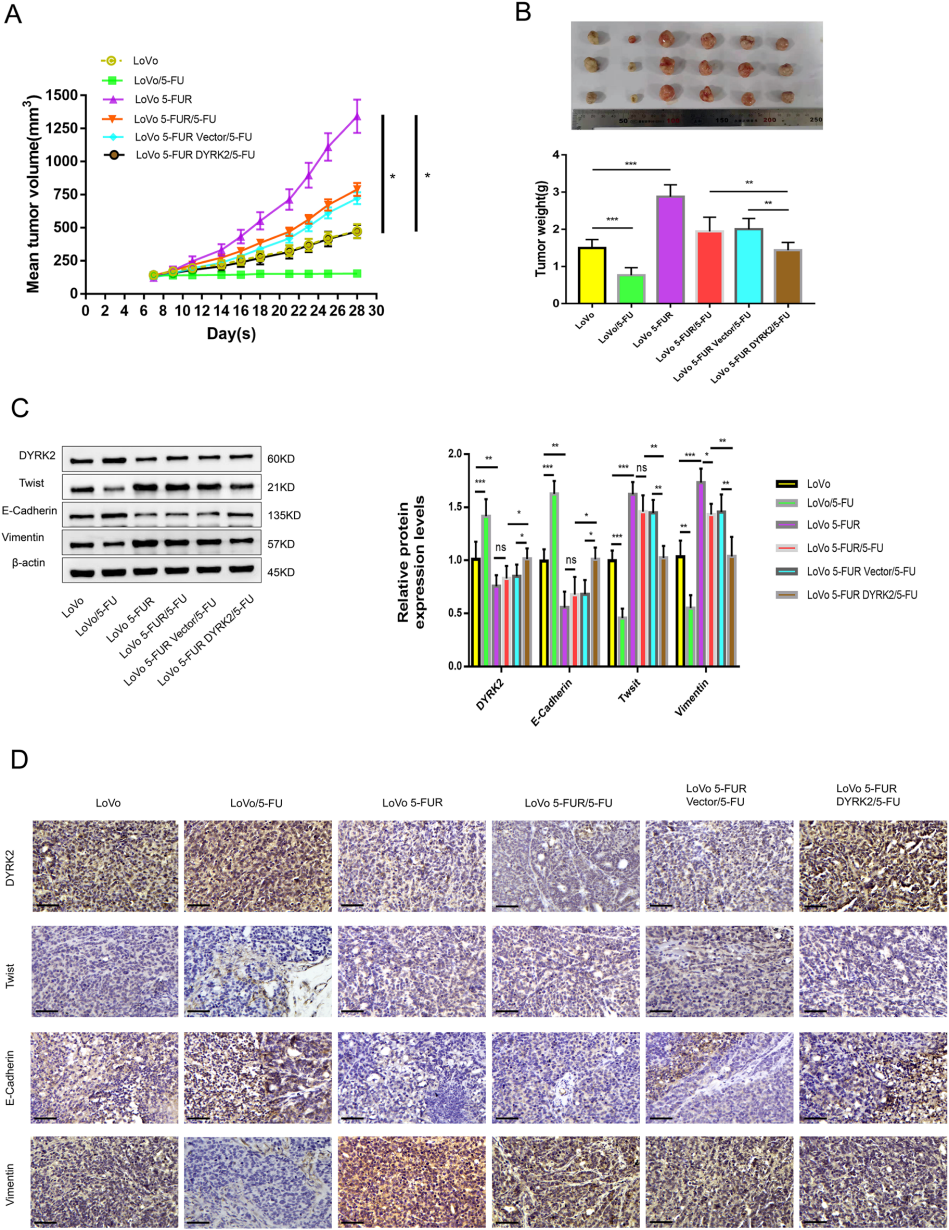
DYRK2 inhibits the in vivo growth of CRC tumors via targeting Twist. (**A**) Changes in subcutaneously implanted tumor volume over time. (**B**) Tumor weight values at the end of the study period in the indicated groups. (**C**) DYRK2, Twist, E-Cadherin, and Vimentin expression were assessed via Western blotting. (**D**) IHC staining was used to detect DYRK2, Twist, E-cadherin, and Vimentin expression in the indicated groups.

Next, animal model experiments were conducted revealing that the overexpression of DYRK2 significantly reduced tumor growth with respect to both tumor volume (Fig. 7A) and tumor weight (Fig. 7B). After 3 weeks of treatment, mice were euthanized and tumor inhibition rates were calculated, revealing significantly more rapid growth for tumors in the 5-FUR LoVo group relative to the LoVo group (p = 0.024), confirming the enhanced malignancy of these chemoresistant cells as observed in vitro*.* When nude mice in the 5-FUR LoVo group were treated with 5-FU, reduced tumor growth was observed, with a calculated 41.39% tumor inhibition rate. The tumor inhibition rate in the 5-FUR LoVo vector/5-FU group was 46.16%, while that for the 5-FUR LoVo DYRK2/5-FU group was significantly increased to 64.32% (p = 0.027). These results confirmed that DYRK2 was able to significantly enhance the chemosensitivity of 5-FUR LoVo cells.

Relative to tumors in nude mice in the LoVo group, those from mice in the 5-FUR LoVo group exhibited lower levels of DYRK2 expression and increased Twist expression, with concomitant decreases in E-cadherin expression and increases in Vimentin expression, consistent with EMT induction in these chemoresistant tumors. Relative to tumors from the 5-FUR LoVo/5-FU group, DYRK2 was upregulated and Twist was downregulated in DYRK2-overexpressing 5-FUR LoVo tumors subjected to 5-FU treatment, with a corresponding inhibition of EMT induction as evidenced by E-cadherin upregulation and Vimentin downregulation. These results thus suggested that DYRK2 may inhibit the EMT in 5-FUR LoVo cells via targeting Twist, thereby regulating tumor growth and chemoresistance (Fig. 7C). IHC staining further confirmed that there were marked increases in DYRK2 and E-Cadherin protein levels in xenograft tumors from the 5-FUR LoVo DYRK2/5-FU group as compared to the 5-FUR LoVo/5-FU group (Fig. 7D). Together, these data confirmed that DYRK2 can regulate tumor growth and EMT induction in part via suppressing Twist expression.

### A combination of DYRK2 and Twist levels offers prognostic utility in CRC patients

**Figure 8:**
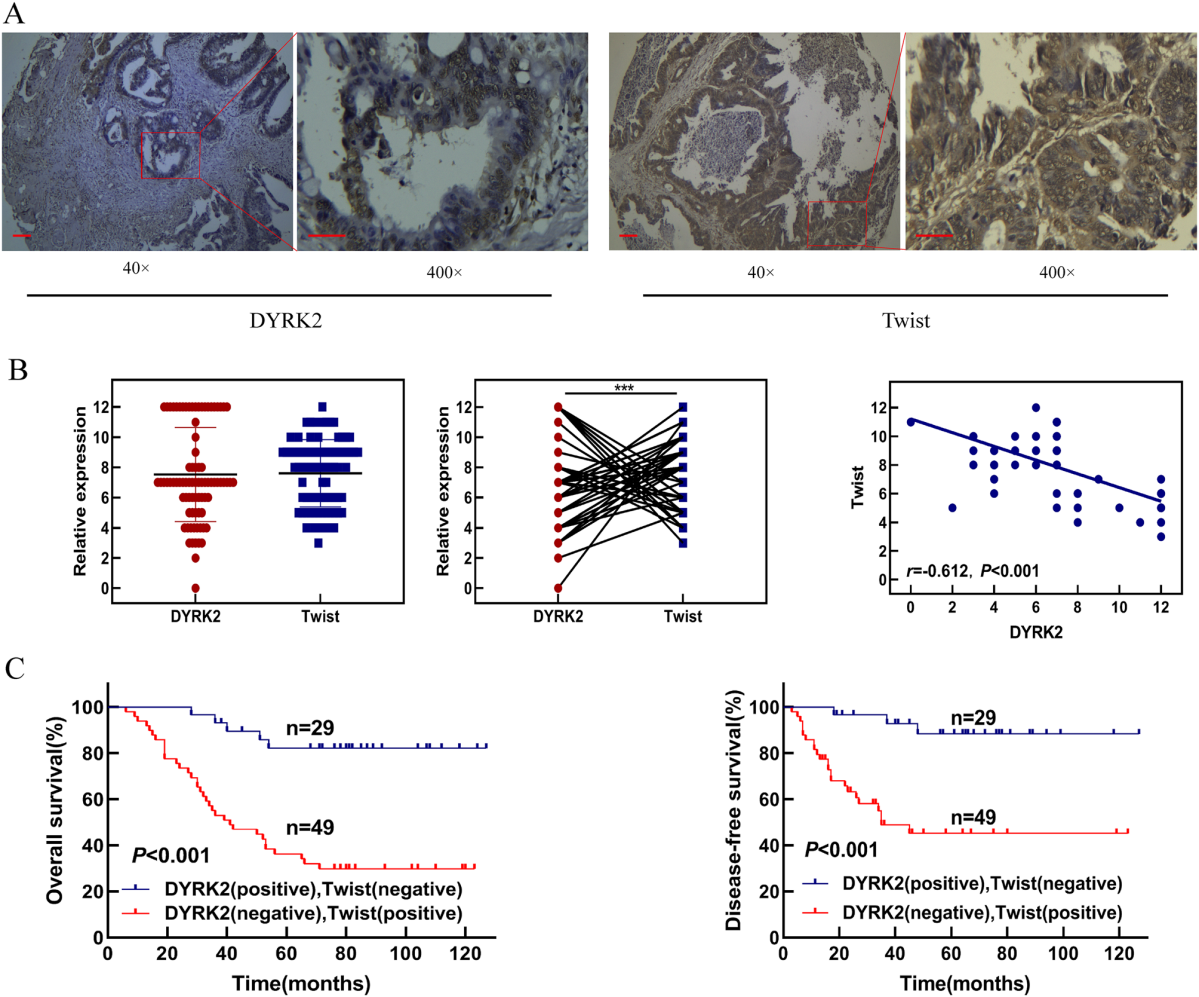
Combination of DYRK2 and Twist levels is useful for predicting clinical prognosis of CRC patients. (**A**) Representative images of IHC staining for DYRK2 and Twist. Note: Scale bar = 100 μm (× 40) or 20 μm (× 400). (**B**) Distribution and correlation analysis of DYRK2 and Twist in CRC samples (r = − 0.612, *p* < 0.001). (**C**) Prognostic values of DYRK2 combined with Twist.

To further verify the correlation between the expression of DYRK2 and Twist in the CRC cases, we investigated the expression of Twist in CRC TMA samples used to analyze DYRK2 expression above. These analyses revealed that Twist expression was evident at high levels in CRC samples in which DYRK2 expression was lower (Fig. 8A). Statistical analysis revealed that expression of Twist was negatively correlated with DYRK2 in CRC tissues (Fig. 8B). Moreover, pairwise comparisons indicated that patients with high DYRK2 and low Twist staining levels had significantly better OS and DFS than patients with low DYRK2 and high Twist staining (Fig. 8C). This indicated that the combination of DYRK2 and Twist levels can offer value as a tool for predicting the clinical prognosis of CRC patients.

## Discussion

Recent studies have explored the mechanistic role of DYRK2 in the context of cellular development, proliferation, and growth, with a particular focus on its oncogenic activity^22,23^. In the present report, we assessed the ability of DYRK2 to influence the chemosensitivity of 5-FUR LoVo cells via modulating the induction of the EMT in these CRC cells, thus increasing the sensitivity of these cells to 5-FU treatment. Through analyses of TMAs and the TCGA database, DYRK2 was found to be downregulated in CRC tumor tissues relative to healthy colorectal tissue samples, with its downregulation being associated with patient clinicopathological characteristics. Relative to patients expressing higher levels of DYRK2, those expressing lower levels of this gene were more likely to exhibit lymph node metastasis, liver metastasis, and worse pathological typing, consistent with DYRK2 downregulation being consistent with a poorer prognosis. Moreover, DYRK2 downregulation was associated with a significantly shorter disease-free survival and overall survival in these CRC patients, potentially as these patients are more likely to develop resistance to 5-FU treatment, resulting in poorer treatment outcomes and higher rates of recurrent or metastatic disease. In a prior study of DYRK2 in CRC, Laham AJ et al. used publicly available data and web-based tools to investigate the status of DYRK expression in CRC by comparing these levels in normal and tumor tissue samples and across multiple stages, molecular subtypes, and histological subtypes of CRC. They found that DYRK2 was the only kinase found to be significantly downregulated in CRC, and they also found that DYRK2 was upregulated in CRC MSS subtypes with stable DNA repair mechanisms^18^. Ito et al. reported for the first time that the overexpression of DYRK2 inhibits CRC liver metastasis, indicating that patients undergoing the resection of CRC liver metastases who have lower levels of DYRK2 may require closer surveillance as compared to those with high levels of DYRK2^20^. In lung cancer, it has been found to be associated with patient prognosis^24^, with similar reports having linked this gene to poorer prognostic outcomes in hepatocellular carcinoma^25^ and breast cancer^26^. Moreover, decreased DYRK2 expression levels have been linked to chemoresistance in bladder cancer^27^.

We further explored the expression of DYRK2 in different colorectal cell lines and 5-FU resistant cells (5-FUR), and observed low levels of DYRK2 expression in CRC 5-FUR lines (LoVo 5-FUR, HCT116 5-FUR, SW480 5-FUR) as compared to parent cells (LoVo, HCT116, SW480) at both the protein and mRNA levels (Fig. 2A). It was obviously that DYRK2 was significantly decreased in LoVo 5-FUR as compared to parental LoVo cells. Therefore, we chose to further study the biological behavior of DYRK2 in LoVo and LoVo 5-FUR cells. While the mechanistic basis for such downregulation remains to be fully clarified, there are two possible explanations for this result. One may be that DYRK2 is downregulated following the acquisition of 5-FU resistance, whereas the other is that DYRK2 may modulate gene expression in a manner conducive to the development of a chemoresistant phenotype. Further research will be necessary to clarify these underlying mechanisms.

We additionally evaluated the impact of DYRK2 as a regulator of the biological activity of 5-FUR LoVo cells. Relative to parental LoVo cells, these chemoresistant cells exhibited more malignant characteristics including enhanced proliferation, reduced chemosensitivity, greater apoptotic resistance, and improved migratory and invasive activity (Fig. 2B–E). Notably, E-cadherin was downregulated in these cells whereas Vimentin was upregulated, consistent with EMT induction in these cells. Such EMT induction is a common phenotype in tumor cells of epithelial origin, with affected cells exhibiting a lack of epithelial E-cadherin and β-catenin marker expression on tumor cell membranes with the concomitant induction of mesenchymal phenotypes characterized by decreased adhesion and enhanced Vimentin expression. Following EMT induction, these cells also acquire greater migratory and invasive activity, enabling their metastatic progression^28–31^.

The EMT procedure is an important mediator of chemoresistance. Shen et al. previously demonstrated the ability of CircFoxo3 to reverse EMT induction in docetaxel-resistant prostate cancer cells, thereby enhancing their chemotherapeutic sensitivity^32^. Cisplatin-resistant epithelial ovarian cancers exhibit EMT-like properties including enhanced migratory and invasive activity, while BEZ235, a dual PI3K/mTOR inhibitor, was able to reverse EMT phenotypes, reversing these invasive and migratory capabilities, restoring their chemotherapeutic sensitivity^33^. Mesenchymal phenotypes are evident in cisplatin-resistant human breast cancer cell lines^34^. Zhang et al. demonstrated that the novel kinase DSTYK promotes both TGF-β-induced EMT and subsequent chemoresistance in CRC cells^9^. Ma et al. found that Lgr5 functions as a tumor promoter that can increase cell migration and promote EMT induction in HCC cells, enhancing their resistance to Doxorubicin^35^.

The results of this study provide novel evidence that DYRK2 can inhibit CRC cell chemoresistance by controlling EMT induction. Such chemoresistance may, in turn, contribute to enhanced malignancy and treatment insensitivity. Given the critical role of the EMT in oncogenic contexts, underscoring the need for further research regarding how different molecules regulate the relationship between the EMT and drug resistance. In the present report, we found DYRK2 overexpression to result in E-cadherin upregulation and concomitant Vimentin downregulation, consistent with the ability of DYRK2 to inhibit EMT induction within these 5-FUR LoVo cells. Moreover, these DYRK2-overexpressing 5-FUR LoVo cells exhibited impaired invasion, proliferation, and migration with concomitant increases in apoptotic cell death and chemosensitivity (Fig. 4B–F), indicating that DYRK2 can suppress the malignancy of these cells owing to its ability to regulate EMT induction.

Given the marked impact of DYRK2 on the induction of the EMT in CRC cells, we next sought to explore the downstream molecular mechanisms. Master transcription factors that regulate the EMT in response to signaling through appropriate cell surface receptors include Twist, Snail, Slug, LFE-1, ZEB1, and TGF-β^36,37^. For example, Ryu et al. explored the effect of Snail phosphorylation on EMT regulation, and found that p38 MAPK is an important post-translational regulator that enhances Snail stability. Analyses of clinical samples have identified a critical role for the p38-Snail axis in the regulation of ovarian cancer EMT and metastasis^38^. We detected the expression of common EMT-transcription factors such as Twist, Snail, ZEB1 in LoVo 5-FUR NC, LoVo 5-FUR Vector, LoVo5-FUR DYRK2 cell lines, respectively. The results showed that Snail and ZEB1 had no significant difference among the three groups, while Twist was highly expressed in LoVo5-FUR NC and LoVo5-FUR Vector group, but relatively low expressed in LoVo 5-FUR DYRK2 group. Therefore, we predicted that DYRK2 might affect the biological behavior of LoVo 5-FUR by regulating Twist. Therefore Twist was chosen for further study. Here, we observed a significant reduction in Twist protein levels when DYRK2 was overexpressed, while MG132 treatment was sufficient to restore these levels in the presence of DYRK2 overexpression. To confirm the impact of DYRK2 on Twist ubiquitination, we conducted Co-IP experiments revealing DYRK2, when overexpressed, to efficiently induce the ubiquitination of Twist. Our data thus confirmed that DYRK2 was able to induce the degradation of Twist via the ubiquitin-mediated proteasome. In animal studies, the overexpression of DYRK2 was associated with impaired tumor growth, in addition to inhibiting Twist and Vimentin expression while enhancing Vimentin expression within CRC xenograft tumors. We further explored the relationship between Twist and DYRK2 expression in CRC patients, revealing that Twist was negatively correlated with DYRK2 in CRC tissues. Patients with high DYRK2 and low Twist staining levels exhibited significantly better OS and DFS than did patients with low DYRK2 and high Twist staining. Taken together, these analyses indicated that the expression of high levels of the EMT regulator DYRK2 resulted in the downregulation of Twist and ultimately suppressed CRC malignancy.

In summary, our data suggest that DYRK2 may represent a novel therapeutic target in CRC. DYRK2 is an important regulator of CRC cell proliferative, invasive, and migratory activity, functioning by regulating Twist and thereby modulating EMT induction, chemosensitivity, and apoptosis in these tumor cells. As such, DYRK2 may offer value as a prognostic biomarker in this cancer type, with CRC patients exhibiting lower levels of DYRK2 expression potentially requiring closer monitoring than patients expressing higher levels of this gene. In addition, DYRK2 may represent a promising therapeutic target in this cancer type, although further studies will be needed to validate this hypothesis.

## Materials and methods

### Patient tissue microarray processing

Human CRC tissue microarray slides consisting of 83 pairs of CRC patient tumor tissue samples were purchased from Shanghai Superbiotek. Samples were initially heated, deparaffinized, boiled in a pressure cooker in a 10 mmol/L sodium citrate (pH 6.0) solution to facilitate antigen retrieval, and probed with primary anti-DYRK2 (NOVUS, USA), and anti-Twist (abcam,UK). Slide staining intensity was scored as in prior reports^39^. 0 points, negative staining; 1 point, weak staining; 2 points, intermediate staining; 3 points strong positive staining. In addition, the frequency of DYRK2-positive cells was scored as follows: 0 points, < 5%; 1 point, 5–25%; 2 points, 26–50%; 3 points, 51–75%; 4 points, > 75%. In data analyses, these scores were multiplied together, with a score of 0–7 or 8–12 being respectively indicative of low or high levels of DYRK2 expression. Clinicopathological characteristics and follow-up data corresponding to these patients were obtained from electronic medical records. All experiments were performed in accordance with relevant guidelines and regulations of the Institutional Ethics Committee of Jiangjin Central Hospital, which approved the present study (20190305-02). Informed consent was obtained from all subjects or their legal guardians.

### Cell culture and 5-FUR CRC cell establishment

The CRC cell line was purchased from ATCC (USA). LoVo, HCT116, SW480, and HCT15 cells were grown in RPMI-1640 (Gibco) containing penicillin/streptomycin (Gibco) and 10% FBS (Gibco). The 5-FU-resistant (5-FUR) LoVo cell line was established as in a previous report^40^. Briefly, LoVo cells were cultured in the presence of gradually increasing 5-FU (Shanxi Pude Pharmaceutical Co., Ltd, China) concentrations over a 6-month period. HCT116 5-FUR, SW480 5-FUR, and HCT15 5-FUR are existing cells in our laboratory, and their culture refers to the culture method of LoVo 5-FUR.The cells were grown in a humidified 5% CO_2_ incubator at 37 °C.

### Lentiviral transduction

A lentiviral vector for DYRK2 overexpression was prepared by Chongqing Genemine Biotechnology Co., LTD. Briefly, primers with restriction sites that were specific for the human DYRK2 mRNA sequences were constructed and used for PCR amplification, yielding the full-length human DYRK2 cDNA. This PC product and the pLenti-CMV vector were then digested with appropriate restriction enzymes and the DYRK2 cDNA was inserted into this pLenti-CMV vector to yield the pLenti-CMV-DYRK2 plasmid, which was subsequently transformed into *E. coli* DH5α that were then screened using ampicillin-containing plates. Positive clones were selected, plasmids were extracted, and restriction enzyme digestion and DNA sequencing were then performed to confirm the sequence identity.

Lentiviral transduction was conducted as reported previously^41^. Briefly, cells (5 × 10^4^/well) were transduced with the DYRK2 or empty control lentiviruses at an appropriate multiplicity of infection in media containing 10% FBS. At 48 h post-transduction, cells were selected using 4 µg/mL blasticidin (Solarbio, China).

### Immunohistochemical and immunofluorescent staining

Immunohistochemical (IHC) staining was conducted as reported previously by Yamadera et al.^42^. Briefly, xenograft tumors were excised from experimental animals, fixed using 4% paraformaldehyde (PFA), dehydrated with an ethanol gradient, deparaffinized, dehydrated, blocked with serum, and probed with appropriate primary antibodies. A DAB kit was used for signal detection, while hematoxylin was used for nuclear counterstaining.

Immunofluorescent staining was conducted as previously reported by Hou et al.^43^. Briefly, cells were fixed, permeabilized, and blocked, followed by incubation with an appropriate primary antibody. Cells were then washed thrice with PBS, incubated with appropriate secondary antibodies, and nuclei were counterstained using DAPI. A fluorescence microscope (Olympus Corporation, Tokyo, Japan) was used to image cells at × 200 magnification.

### Cell proliferation analyses

A Cell Counting Kit-8 (CCK-8; MedChemExpress) was used to assess cellular proliferation and viability. Briefly, cells were treated with 5-FU (0, 0.078, 0.156, 0.312, 0.625, 1.25, 2.5, 5, or 10 µg/mL) for 24, 48, or 72 h following transduction with DYRK2-overexpression or empty control lentiviruses. Absorbance at 450 nm was then assessed via microplate reader (Thermo Fisher Scientific).

### Apoptosis analysis

An Annexin V-FITC Apoptosis Detection Kit (BD Pharmigen) was used to evaluate the apoptotic death of CRC cells. Briefly, cells (5 × 10^5^) were collected following appropriate treatment and suspended in 1 × binding buffer, after which they were stained for 5 min with Annexin V-FITC and propidium iodide (PI) while protected from light at room temperature. Cells were then imaged via flow cytometry, with the percentage of apoptotic cells being determined based upon the number of cells in the early and late phases of apoptotic death. Data were captured using a CytoFLEX flow cytometer and analyzed with the CytExpert 2.4 software (Beckman Coulter, Inc.).

### Transwell assays

Transwell inserts (8.0 µm pore size, Corning, USA) were used to assess cellular migration and invasion in 24-well plates. Briefly, 1 × 10^5^ cells were added to the upper chamber of Transwell inserts that were either uncoated or coated with Matrigel (Corning, USA) in 200 µL of serum-free media to assess migratory or invasive activity, respectively. The lower chamber in each well was then filled with 600 µL of culture media containing 5% FBS, and cells were incubated for 24 h. The cells remaining on the upper surface of the Transwell insert were carefully removed, while the remaining cells were fixed and stained for 15 min using 1% crystal violet. Cells were then counted via light microscopy. All analyses were performed in triplicate.

### Quantitative real-time PCR

TRIzol (TAKARA, Japan) was used to extract total RNA from cells, after which a NanoDrop spectrophotometer (Thermo Fisher Scientific, Inc.) was used to measure RNA concentrations. All quantitative real-time PCR (qPCR) analyses were performed based on provided directions. The expression of Twist and DYRK2 was assessed via qPCR using primer sets obtained from Sangon Biotech Co., Ltd. Relative gene expression was assessed via the 2^−ΔΔCT^ method^44^. Utilized primers are as follows: DYRK2-F: 5′-GAAGCGCCAGGGCATGACAG-3′, DYRK2-R: 5′-CGCAGGTGTTCCAGGATTCG-3′; Twist-F: 5′-GCGGCCAGGTACATCGACTTC-3′, Twist-R: 5′-GCCCCCTCCATCCTCCAGAC-3′; β-actin-F: 5′-ACCCCGTGCTGCTGACCGAG-3′, β-actin-R: 5′-TCCCGGCCAGCCAGGTCCA-3′.

### Western blotting and co-immunoprecipitation

Western blotting and co-immunoprecipitation (Co-IP) were performed as in a previous study^45^. Briefly, protein lysates were prepared from cells, separated via SDS-PAGE, and transferred onto PVDF membranes that were subsequently blocked using 5% non-fat milk and probed overnight at 4 °C with antibodies specific for DYRK2 (Novus, USA), Twist (abcam, UK), E-cadherin (CST, USA), Vimentin (CST, USA), Ubiquitin (CST, USA), Snail (CST, USA), ZEB1 (abcam, UK) and β-actin (SC, USA). Blots were then incubated with secondary antibodies. MG132 (CST, USA) was added as experimentally appropriate.

LoVo 5-FUR NC, LoVo 5-FUR Vector, and LoVo 5-FUR DYRK2 cells were used to prepare lysates for Co-IP analyses, which were then pre-cleared via incubation with protein A + G Agarose (Beyotime, China). Bound proteins were then separated via SDS-PAGE, with antibodies specific for Twist, or Ubiquitin being added to these lysates.

### In vivo xenograft model experiments

Male athymic mice (nu/nu; n = 30) were purchased from Beijing Huafukang Biotechnology Co., Ltd. [certificate. SCXK(JING)2019-0008]. Laboratory Animal Welfare and Ethics Committee of Chongqing University approved all animal investigations, and all methods were carried out in accordance with the relevant guidelines and regulations. Mice were randomly assigned to 6 experimental groups (n = 5/group) at 4 weeks of age, and were subcutaneously implanted with appropriate tumor cells (50 µL at 3 × 10^6^/mL) in the right armpit. At 7 days post-implantation, mice were intraperitoneally treated with 5-FU (20 mg/kg every other day for 21 days). On day 29, mice were humanely euthanized via the injection of 1% sodium pentobarbital at a concentration of 100 mg/kg. Tumor volume was calculated as follows: 1/2 × a × b^2^, where a and b respectively correspond to the longest tumor diameter and the tumor diameter perpendicular to a. At the end of the study period, tumor tissues were harvested from these mice. The tumor inhibition rate was calculated as follows: Tumor inhibition rate = (Tumor Volume_control_ − Tumor Volume_experimental_)/Tumor Volume_control_ × 100%. Studies involving animals were performed in accordance with the recommendations in the ARRIVE guidelines.

### Statistical analysis

SPSS 23.0 (IBM Corp., USA) was used to analyze data, which are given as the mean ± SD. Data were compared via unpaired Student’s *t* tests or one-way ANOVAs with Dunnett’s test or Bonferroni post hoc tests as appropriate. The Kruskal–Wallis test was used to analyze data that did not conform to a normal distribution. Categorical data were analyzed via χ^2^ tests or Fisher's exact test. Patient survival outcomes were analyzed via the Kaplan–Meier method. The correlation between DYRK2 and Twist expression was examined by Spearman’s correlation test. P < 0.05 was the threshold of significance.

### Ethic statement

The Institutional Ethics Committee of Jiangjin Central Hospital of Chongqing approved the current exploration (20190305-02). Laboratory Animal Welfare and Ethics Committee of Chongqing University approved all animal investigations, which were in agreement with the Guide for the Care and Use of Laboratory Animals (CQU-IACUC-RE-202203-003).

## Supplementary Information

Supplementary Figures.

## Data Availability

The datasets generated and analysed during the current study are available in its supplementary information files.
