# Supplementary material for: DYRK2 downregulation in colorectal cancer leads to epithelial–mesenchymal transition induction and chemoresistance
Source: Sci Rep. 2022 Dec 28;12:22496. doi: 10.1038/s41598-022-25053-0 (PMC9797492; doi:10.1038/s41598-022-25053-0)
Supplement: Supplementary file 1 — Supplementary Figures. [file 41598_2022_25053_MOESM1_ESM.pdf]

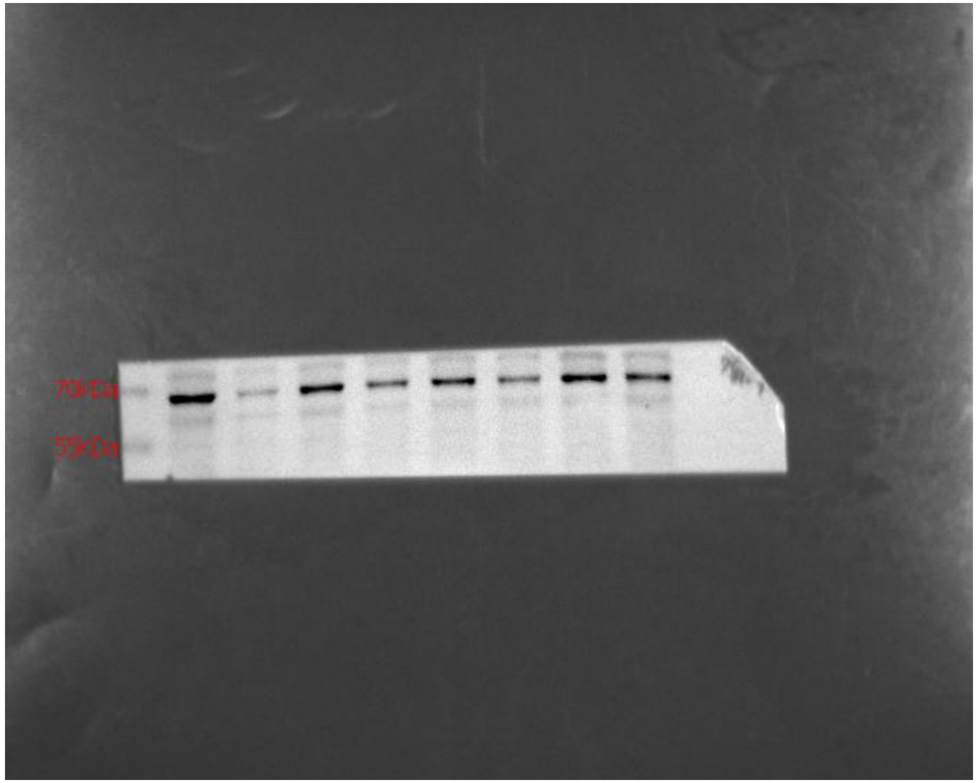

DYRK2

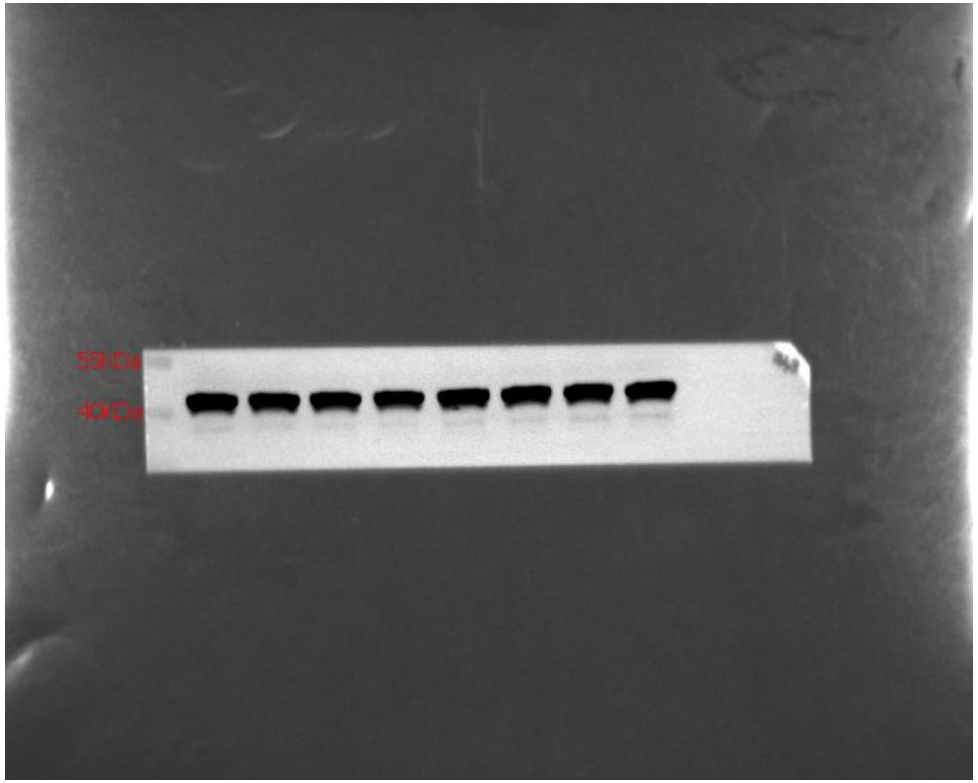

β-actin

Figure 2A

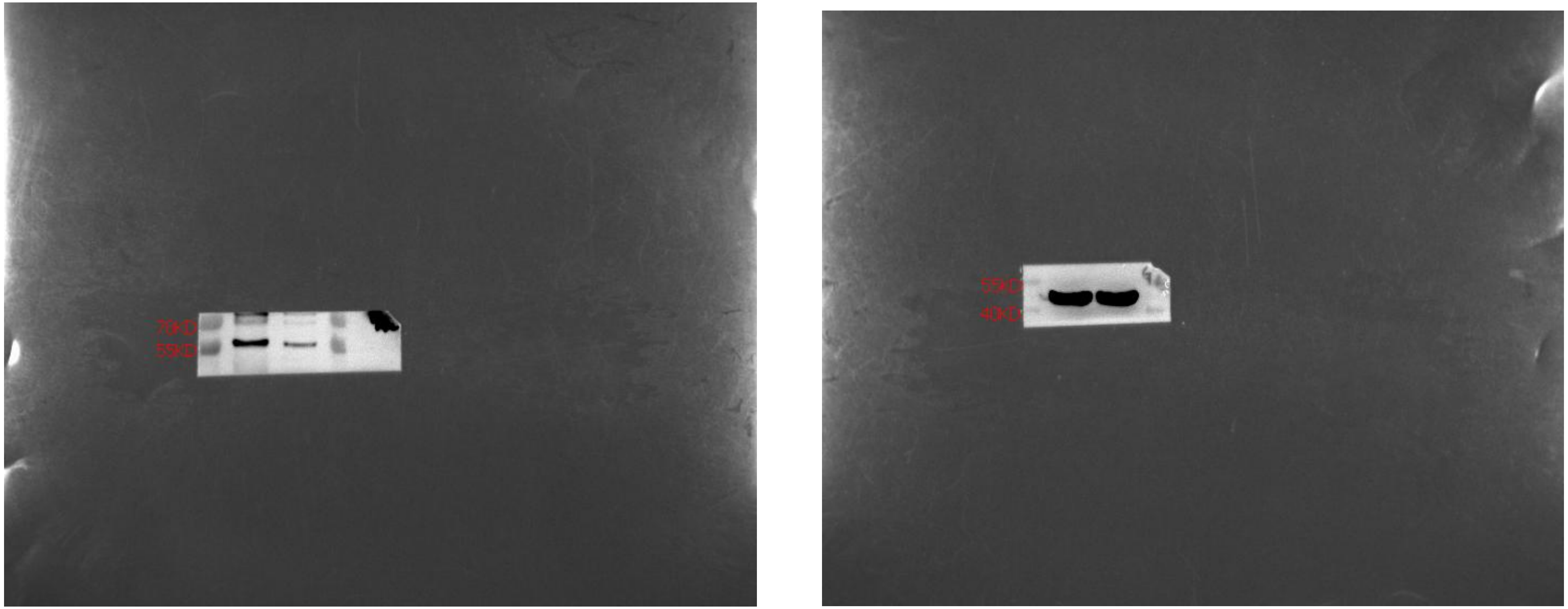

DYRK2

β-actin

Figure 2B

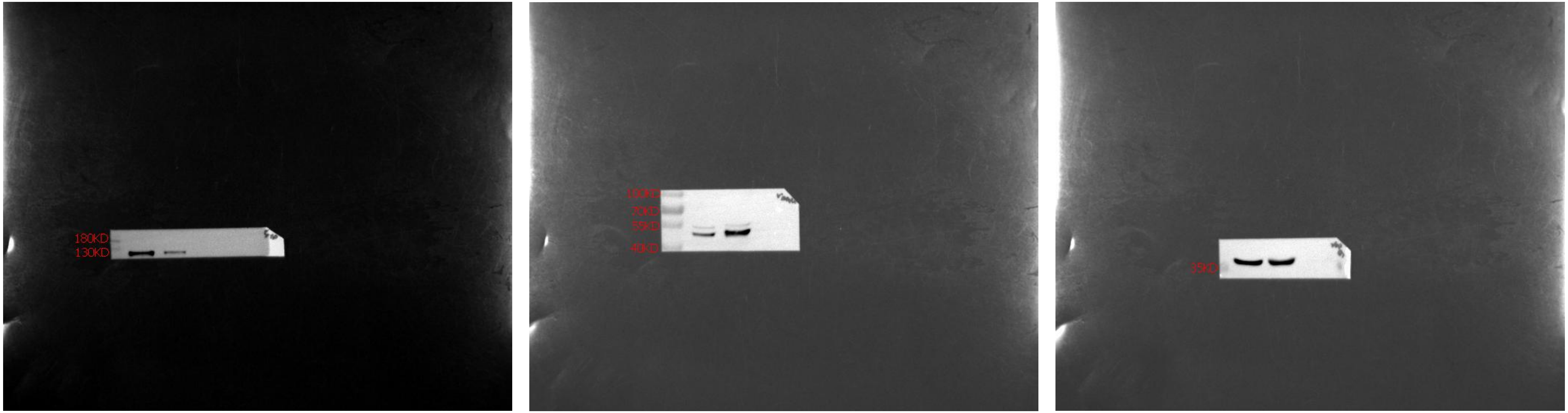

E-cadherin Vimentin β-actin

Figure 3C

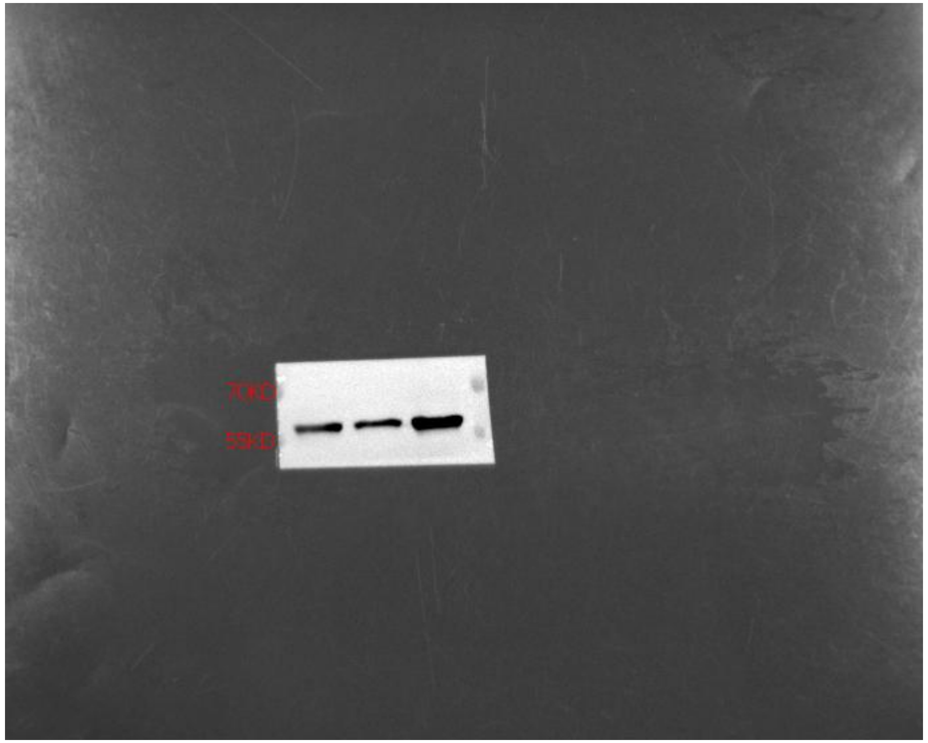

DYRK2

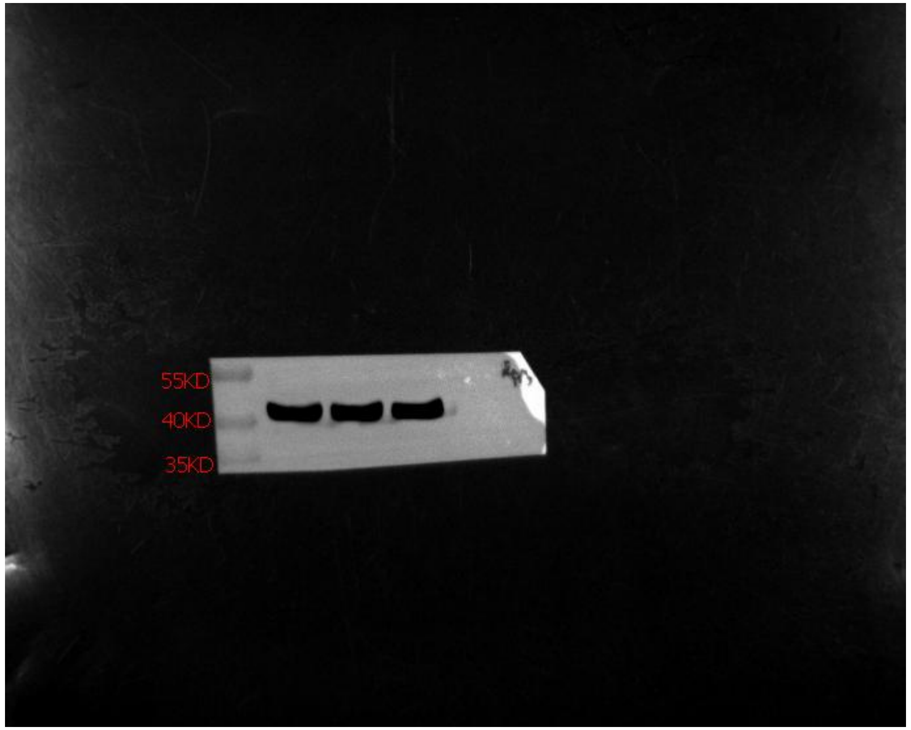

β-actin

Figure 4A

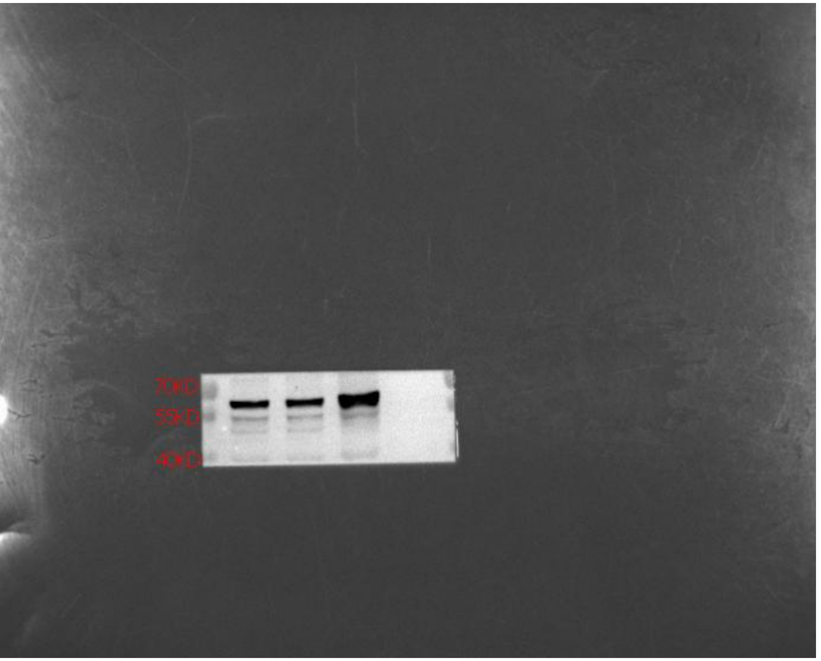

DYRK2

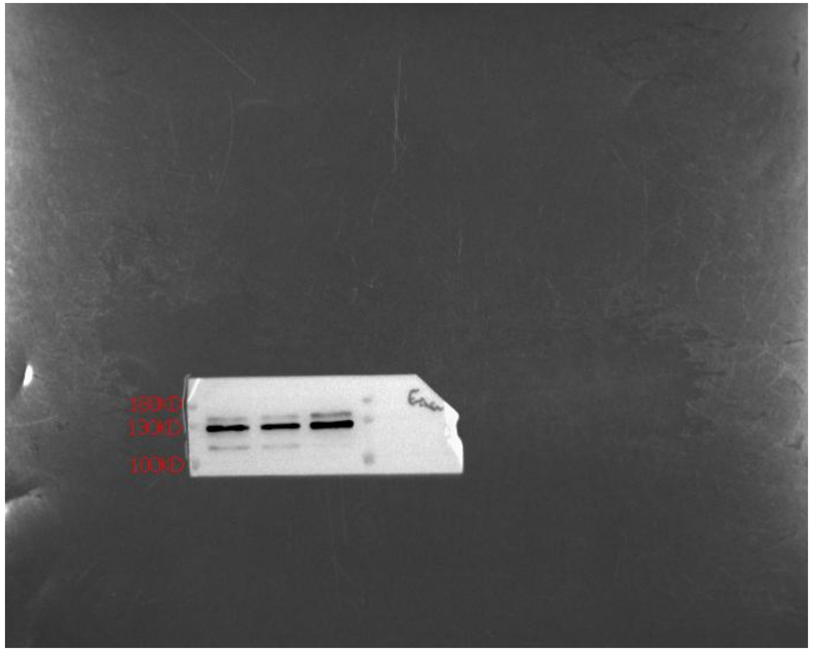

E-cadherin

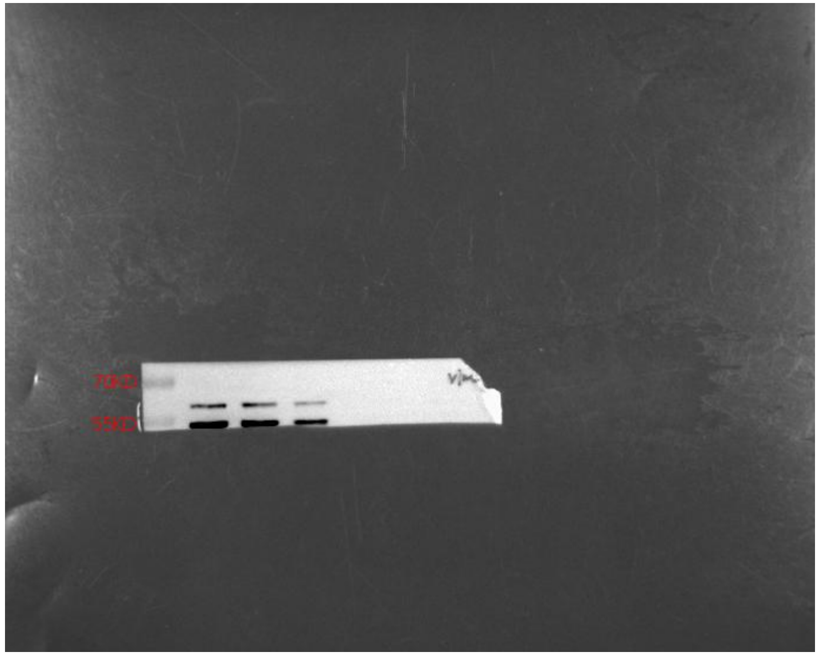

Vimentin

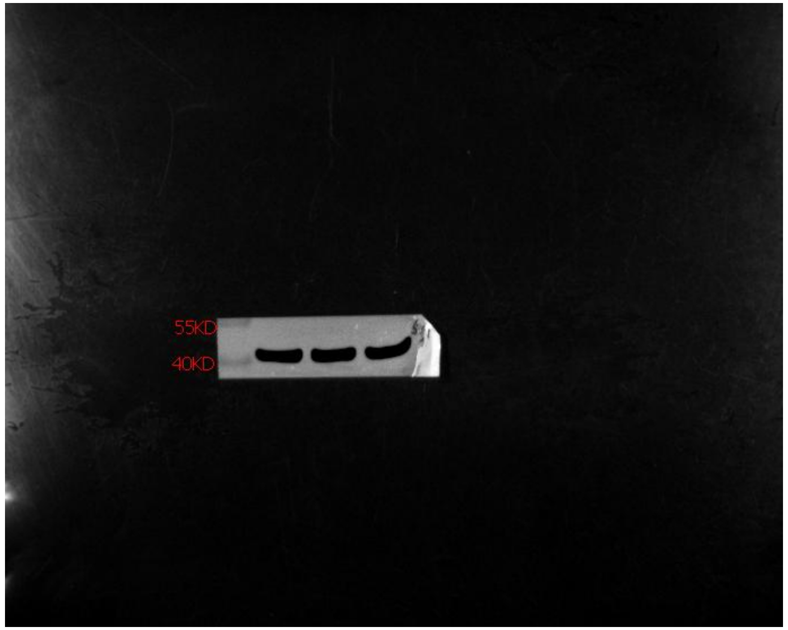

β-actin

Figure 5C

Figure 6A

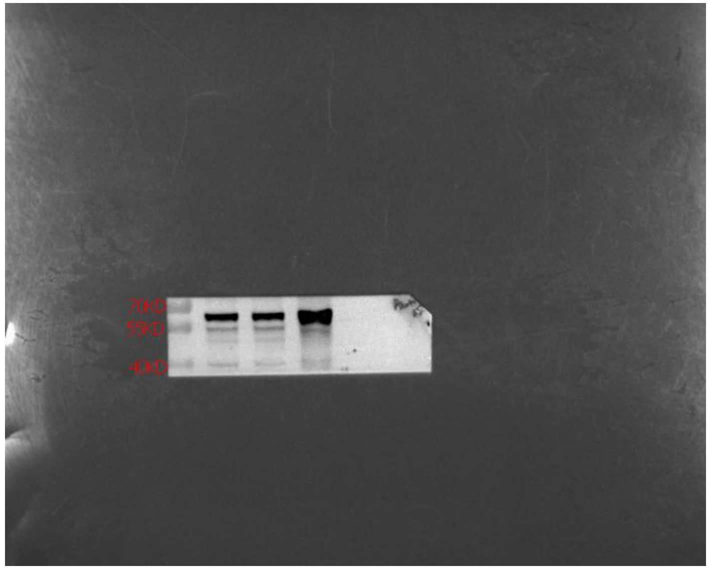

DYRK2

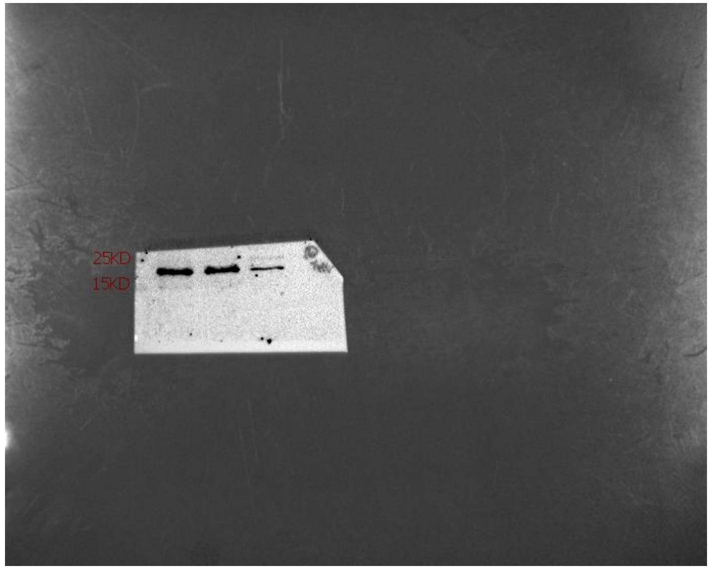

Twist

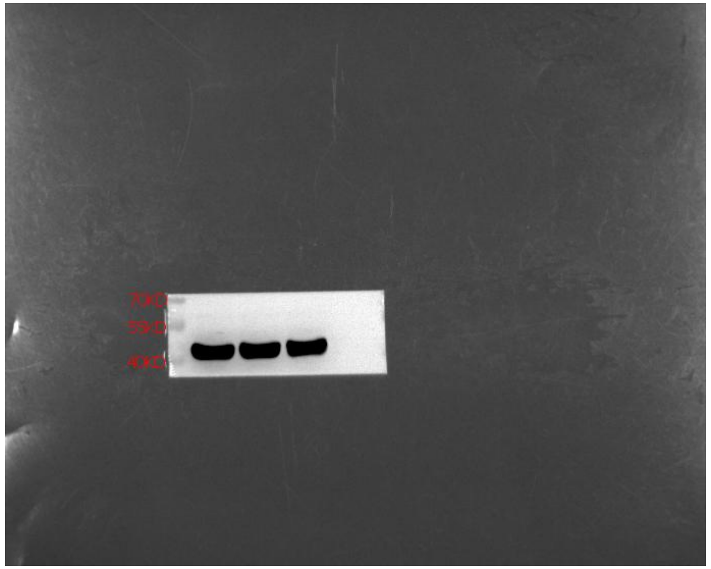

β-actin

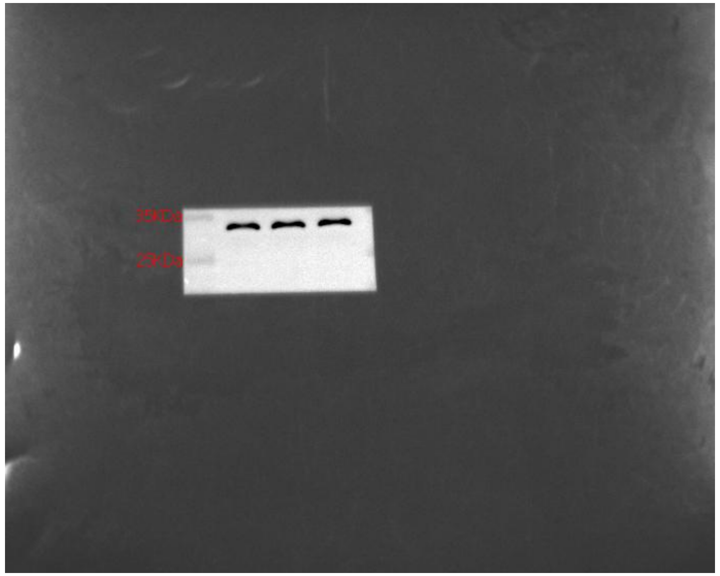

Snail

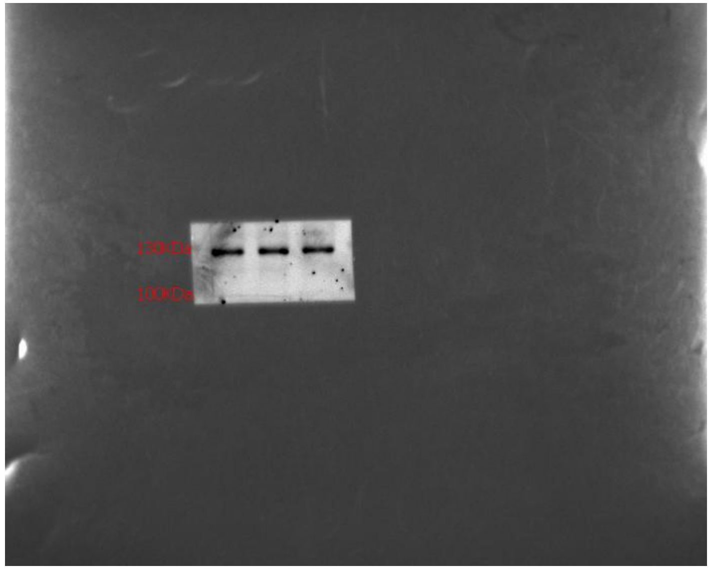

ZEB1

Figure 6C

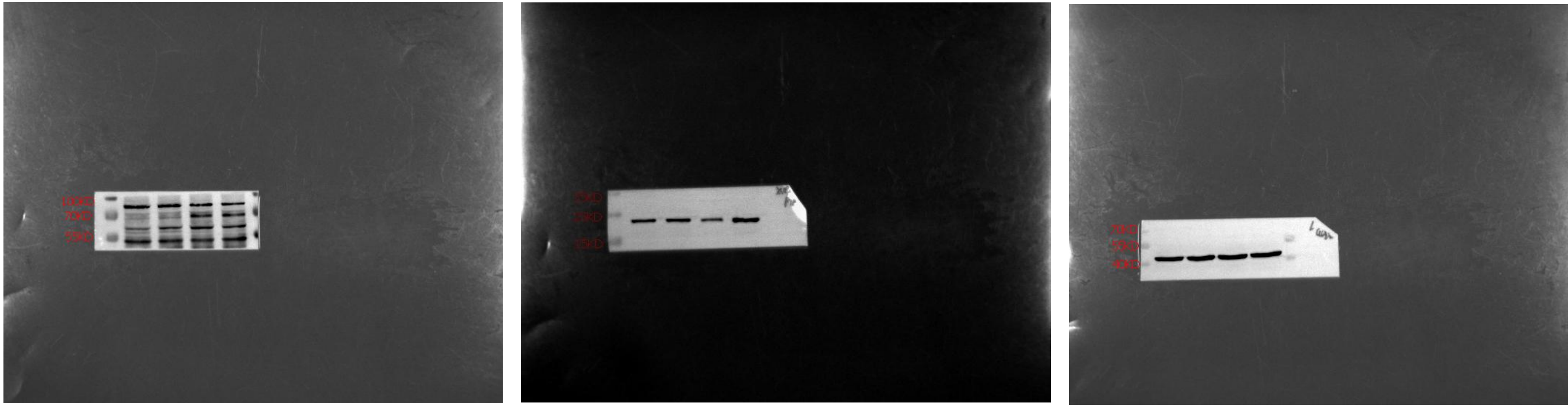

DYRK2 Twist β-actin

Figure 6B

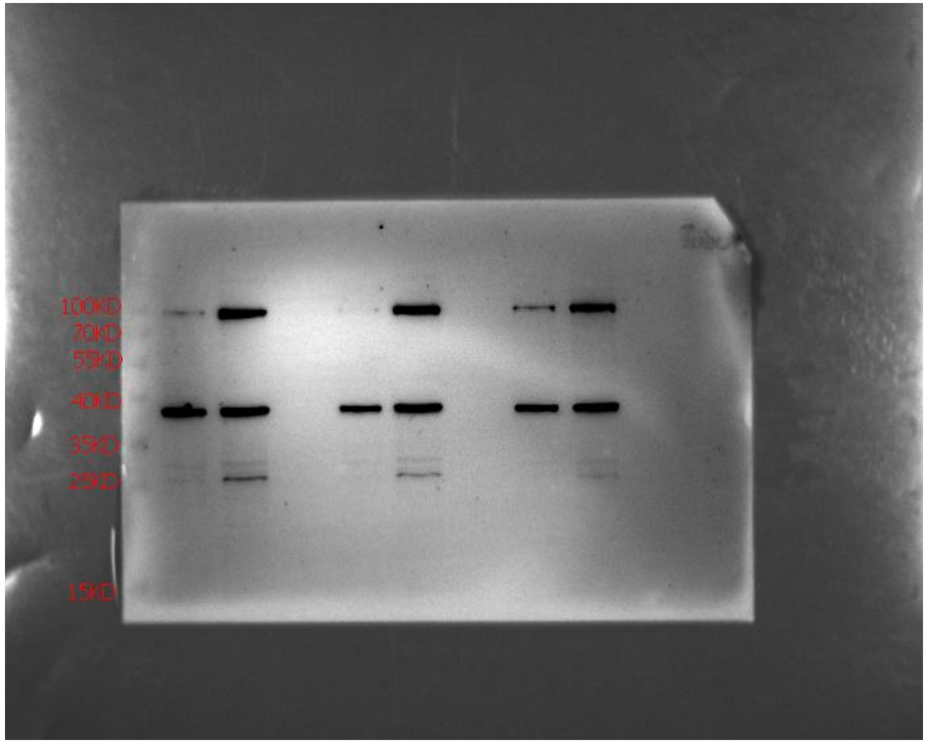

Twist

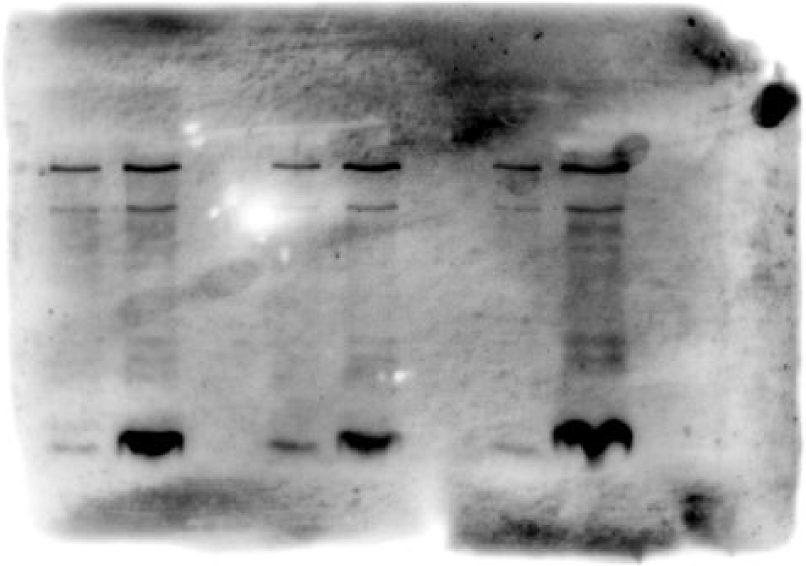

Ubiquitin

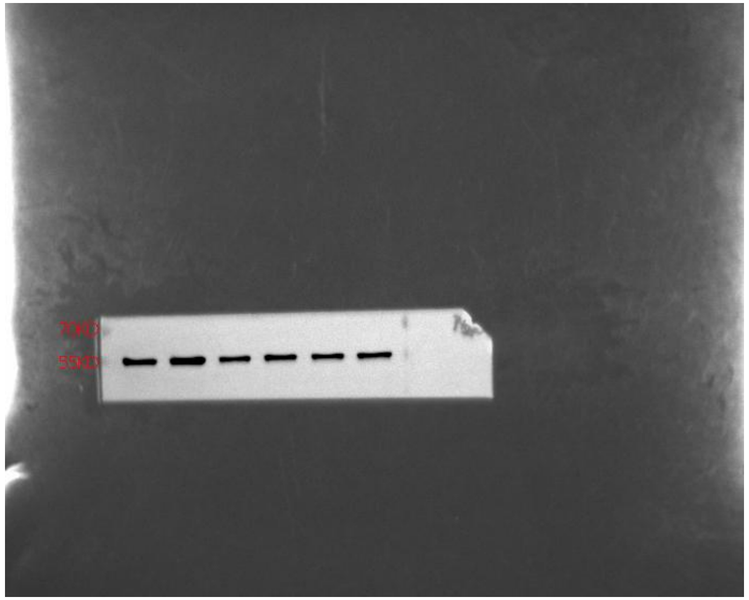

DYRK2

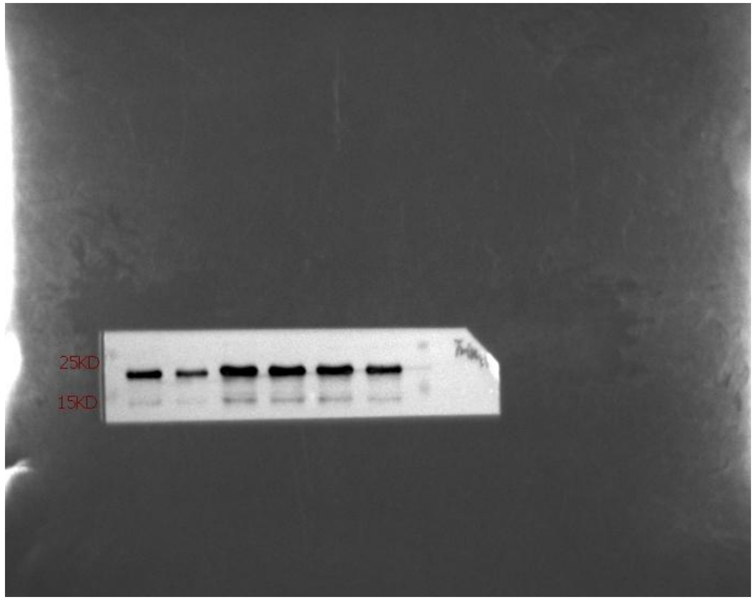

Twist

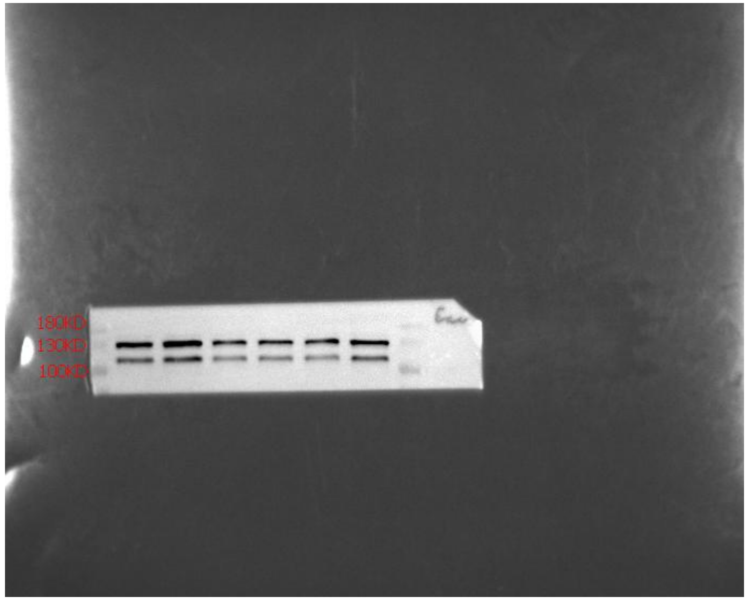

E-cadherin

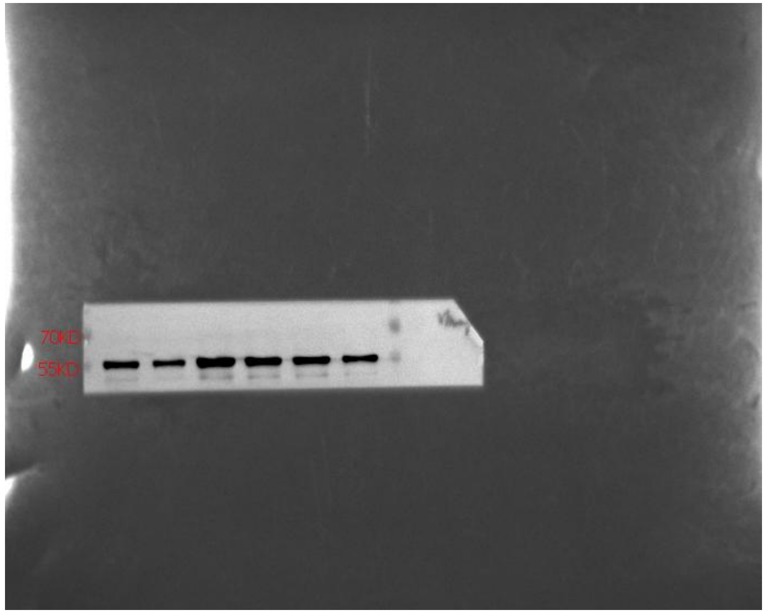

Vimentin

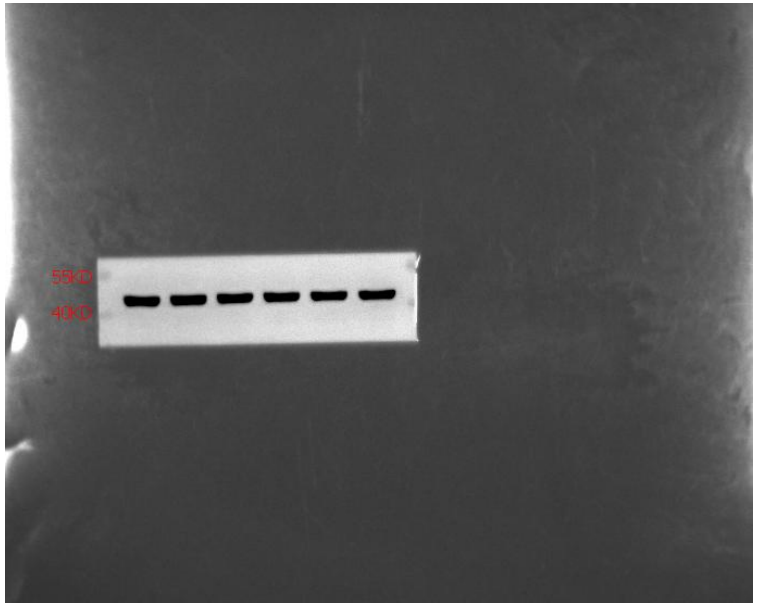

β-actin

Figure 7C
